# Multifunctional PVA composite pads enabled by carvacrol-loaded ZIF-8@PAN microspheres: sustained carvacrol release and long-term blueberry preservation

**DOI:** 10.1016/j.fochx.2026.104460

**Published:** 2026-09-18

**Authors:** Rui Liu, Yu Cheng, Yuanwei Peng, Xuelin Fan, Songzhi Xie, Baohua Liu, Rui Qi, Li Yang, Chun Zhang, Hesheng Xia, Jian Wang

**Affiliations:** a College of Food and Biological Engineering, Chengdu University, Chengdu 610106, China; b State Key Laboratory of Polymer Materials Engineering, Polymer Research Institute, Sichuan University, Chengdu 610065, China; c Institute for Advanced Study, Chengdu University, Chengdu 610106, China

**Keywords:** Polyvinyl alcohol, Polyacrylonitrile microspheres, Carvacrol, ZIF-8, Sustained release, Blueberry preservation

## Abstract

To achieve long-term sustained release of active antibacterial substances and prolong food shelf life, polyacrylonitrile microspheres (PANAM) loaded with ZIF-8 and carvacrol (CV) were fabricated and then blended with crosslinked PVA matrix to prepare a composite pad (PANAM@ZIF-8@CV/PVA). SEM, XPS and XRD results showed ZIF-8 and CV were successfully immobilized in PANAM's porous structure, which effectively enriched active sites and realized efficient anchoring of CV. The triple synergy of ZIF-8, PANAM and polymer matrix enables long-term CV release. Compared with other PVA composite pads containing the same CV content, PANAM@ZIF-8@CV/PVA presented a superior long-term slow-release effect and thus exhibited excellent antioxidant and long-lasting antibacterial properties after 30 days of storage. During 15-day blueberry storage, this composite pad reduced respiratory intensity and weight loss while preserving firmness and nutrients. Thus, blueberries retained intact morphology and fresh taste, demonstrating great potential of PANAM@ZIF-8@CV/PVA in long-term blueberry preservation.

## Highlights

• PANAM loaded with ZIF-8 and CV was successfully synthesized.
• PANAM@ZIF-8@CV were mixed with crosslinked PVA to prepare the composite pad.
• The triple synergistic effect realized long-term sustained release of CV.
• PANAM@ZIF-8@CV/PVA presented excellent antioxidant and antibacterial properties.
• The PANAM@ZIF-8@CV/PVA composite can realize the long-term blueberry preservation.

## Graphical abstract

**Figure:**
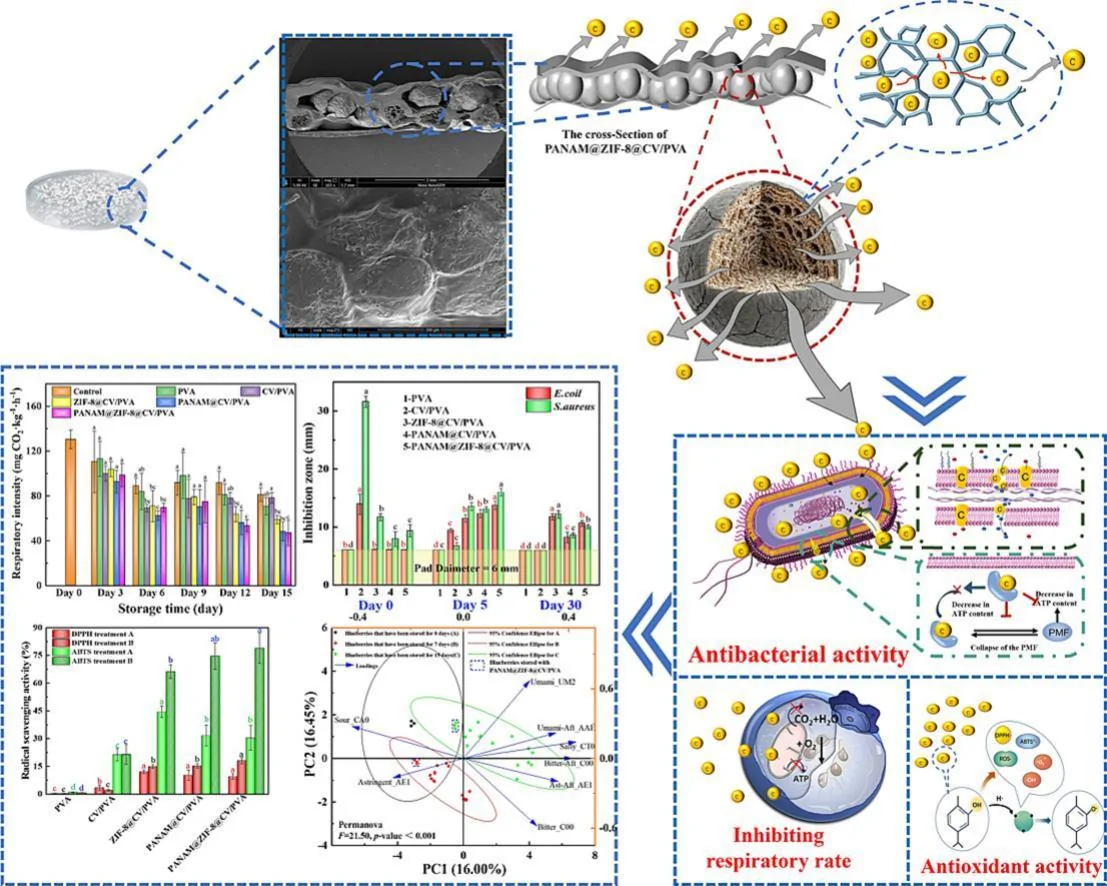

## 1 Introduction

With the continuous advancement of the food industry, the market has imposed increasingly stringent requirements on food packaging materials. Food packaging materials featuring safety, long-term freshness retention and multi-functionality conform to the standards of modern food packaging, especially for perishable foods such as certain fruits and meats that demand preservation during long-haul transportation (Karnwal et al., 2025; Li et al., 2026). Incorporating various reinforced nanofillers into polymer matrices is an effective strategy for preparing such food packaging materials (Wang, Wang, et al., 2025; Wang, Zhou, et al., 2025a). Similarly, incorporating active components with prominent antibacterial and antioxidant properties is another effective approach (Chu et al., 2025; Wang, Zhou, et al., 2025b). These approaches can optimize the overall properties of food packaging materials and further broaden their practical application in the food packaging sector.

Carvacrol (CV) is one of the most widely used natural plant-derived phenolic active components in the food packaging industry. As a monoterpenoid phenol, it serves as a core active ingredient for active food packaging owing to its broad-spectrum antibacterial activity, low toxicity, high biocompatibility and natural source (Babayev et al., 2023; Deng et al., 2024). The food preservation mechanism of carvacrol is based on its gradual release within packaging systems or onto food surfaces, thereby inhibiting microbial proliferation (Listyaningrum et al., 2026). Nevertheless, pure carvacrol suffers from high volatility and short-term preservation effects. Hence, achieving its controlled and sustained release is critical to realize long-duration food preservation (Rzayeva et al., 2025; Xu et al., 2023). Loading carvacrol into MOFs or microcapsules has proven to be an effective strategy to prolong its release behavior.

Metal-organic frameworks (MOFs) are widely employed porous nanofillers assembled through the bonding interaction between metal clusters and assorted organic ligands, with typical representatives including MOF-5, HKUST-1, and ZIF-8 (Jafarzadeh et al., 2024; Khan et al., 2026). On account of their large specific surface area and controllable structure, MOFs are commonly incorporated into other reinforcing fillers for advanced food packaging (Zhang, Zheng, et al., 2022; Zhao et al., 2020). Therefore, combining the unique structural advantages of MOFs with active substances such as carvacrol has emerged as a research hotspot. Encapsulating active agents within MOF matrices to fabricate functional composites exhibits great application potential. The effective combination of MOFs' structural features and bio functional properties with the antibacterial and antioxidant capacities of carvacrol overcomes the limitations of direct carvacrol incorporation (Deng et al., 2024; Khan et al., 2026; Li et al., 2023; Li et al., 2025). Li et al. (2023) pioneered the fabrication of carvacrol-loaded ZIF-8 nanoparticles, which were later mixed with fish scale-derived gelatin and sodium alginate for constructing composite film materials. Immobilization of CV into ZIF-8 imparts the films with sustained carvacrol release and long-term antibacterial performance, extending the shelf life of strawberries to 8 days. This demonstrates the great potential of CV@MOF composite films in food preservation.

Microcapsules are micron-sized miniature carriers that encapsulate solids, liquids or gases within semi-permeable or sealed polymer shells to achieve controlled and sustained release. Further encapsulation of essential oils, carvacrol, and even carvacrol-loaded MOFs into microcapsule-like structures exerts a prominent promoting effect on the sustained release of carvacrol (Deng et al., 2026; Gao et al., 2025; Sun et al., 2021). Therefore, constructing a three-dimensional microcapsule-like structure to immobilize CV@MOF is an effective strategy to improve the sustained-release effect of carvacrol. This three-dimensional structure needs to meet the following requirements: 1. Possess abundant binding sites for high loading capacity of CV@MOF; 2. Contain appropriate porous structures to facilitate mass transfer during the growth of CV and MOFs; 3. Equip with an outer shell to hinder the rapid diffusion of carvacrol from CV@MOF and thereby realize sustained release. Among diverse natural structures, the honeycomb architecture features hierarchically ordered internal pores, which satisfy the mass transfer demands during CV and MOF loading. Meanwhile, its smooth outer shell can restrict the diffusion of carvacrol, perfectly meeting the above requirements. Moreover, fabricating three-dimensional capsule-like microspheres with honeycomb structures from polymers can provide excellent barrier properties and offer abundant active sites for adsorption, loading and interfacial reactions.

In addition, polyvinyl alcohol (PVA) possesses outstanding comprehensive properties including biodegradability, non-toxicity and favorable processability, thus serving as an ideal candidate for food packaging (Daeialiakbar et al., 2025; Hidayaty et al., 2026). By adopting PVA as the matrix, composite materials filled with MOF/carvacrol-loaded polymer microspheres can be fabricated, which exhibit great application prospects in active food packaging and realize the sustained, efficient and controlled release of CV.

Therefore, polyacrylonitrile aerogel microspheres (PANAM) were used to immobilize ZIF-8 — a classic metal-organic framework (MOF) with superior thermal stability, aqueous stability, acid-base resistance, and favorable biocompatibility imparted by zinc ions — as well as CV (PANAM@ZIF-8@CV). The resulting composite was further incorporated into the PVA matrix, with calcium chloride and sodium alginate (SA) serving as crosslinkers, thereby fabricating multifunctional food packaging pads (PANAM@ZIF-8@CV/PVA) with sustained release performance for CV. In the PVA system, the ionic crosslinking network constructed by SA and calcium ions not only efficiently encapsulated and immobilized PANAM@ZIF-8@CV but also preserved more active sites and utilized zinc ions or hydrogen bonding interactions to anchor CV. Such a triple regulatory effect from ZIF-8, PANAM, and the polymer matrix synergistically enables the long-term sustained release of CV. Compared with conventional CV-incorporated PVA materials, the newly developed pads exhibit significant advantages and great potential for applications in long-acting active food packaging.

## 2 Materials and methods

### 2.1 Materials

Zinc nitrate hexahydrate was obtained from Chengdu Jinshan Chemical Reagent Co., Ltd. (Sichuan, China). Polyacrylonitrile (PAN), 2-methylimidazole and carvacrol were supplied by Adamas-beta Co., Ltd. (Shanghai, China). Sodium alginate, polyvinyl alcohol (PVA), and plate count agar were purchased from Shanghai Aladdin Biochemical Technology Co., Ltd. and Beijing Aoboxing Bio-Tech Co., Ltd. (China), respectively. Sodium hydroxide (NaOH), oxalic acid (H_2_C_2_O_4_), anhydrous ethanol, dimethyl sulfoxide (DMSO), methanol and barium chloride were provided by Chengdu Kelong Chemical Reagent Co., Ltd. (Sichuan, China). In addition, 2,2′-azino-bis (3-ethylbenzothiazoline-6-sulfonic acid) diammonium salt (ABTS), l-ascorbic acid (VC), 2,6-dichloroindophenol and 2,2-diphenyl-1-picrylhydrazyl (DPPH) were all purchased from Macklin Biochemical Co., Ltd., Shanghai, China.

### 2.2 Synthesis of different fillers

PAN aerogel microspheres (PANAM) were prepared via electrospraying combined with a solvent exchange strategy. Specifically, PAN was dissolved in DMSO at a concentration of 3 wt%, loaded into a 10 mL syringe, and electrosprayed at 5.2 kV. The collection bath contained a 1:1 *w*/w ethanol/deionized water mixed solvent. After spraying, PAN/DMSO solution droplets dropped into the bath, after which solvent exchange occurred between DMSO and the ethanol/water mixture. Since PAN is insoluble in the ethanol/water system, it formed an insoluble concentrated phase, i.e., PANAM. The obtained PANAM was sieved through a 200-mesh sieve, rinsed three times with deionized water, pre-frozen, and freeze-dried for 48 h.

PANAM@ZIF-8@CV (polyacrylonitrile microspheres loaded with ZIF-8 and carvacrol) was synthesized through a combination of in-situ growth and immersion methods. Briefly, 1.17 g of zinc nitrate hexahydrate was first dispersed in 90 mL of deionized water under gentle stirring. Afterwards, 0.1 g of PANAM was added into the homogeneous zinc nitrate solution, and the mixed system was stirred at room temperature for 4 h to achieve adequate interfacial interaction. Meanwhile, 22.7 g of 2-methylimidazole was added to 80 mL deionized water and dissolved to yield a clear aqueous solution, which was rapidly poured into the above PANAM/zinc nitrate dispersion. The resulting mixture was continuously stirred for another 2 h to promote the in-situ nucleation and growth of ZIF-8 on PANAM. The harvested PANAM@ZIF-8 precipitate was filtered through a 200-mesh nylon sieve, rinsed thoroughly with deionized water 4–5 times to eliminate residual contaminants, and finally collected. The purified and vacuum freeze-dried PANAM@ZIF-8 was soaked in 1% (*w*/*v*) CV methanol solution and magnetically stirred at 100 rpm for 2 h to complete CV loading. After washing with a small amount of methanol to strip loosely bound CV, the product was vacuum freeze-dried for 24 h to acquire the final PANAM@ZIF-8@CV filler. Fig. 1a gives detailed preparation process. In addition, PANAM@CV was fabricated by the identical immersion procedure.

**Fig. 1:**
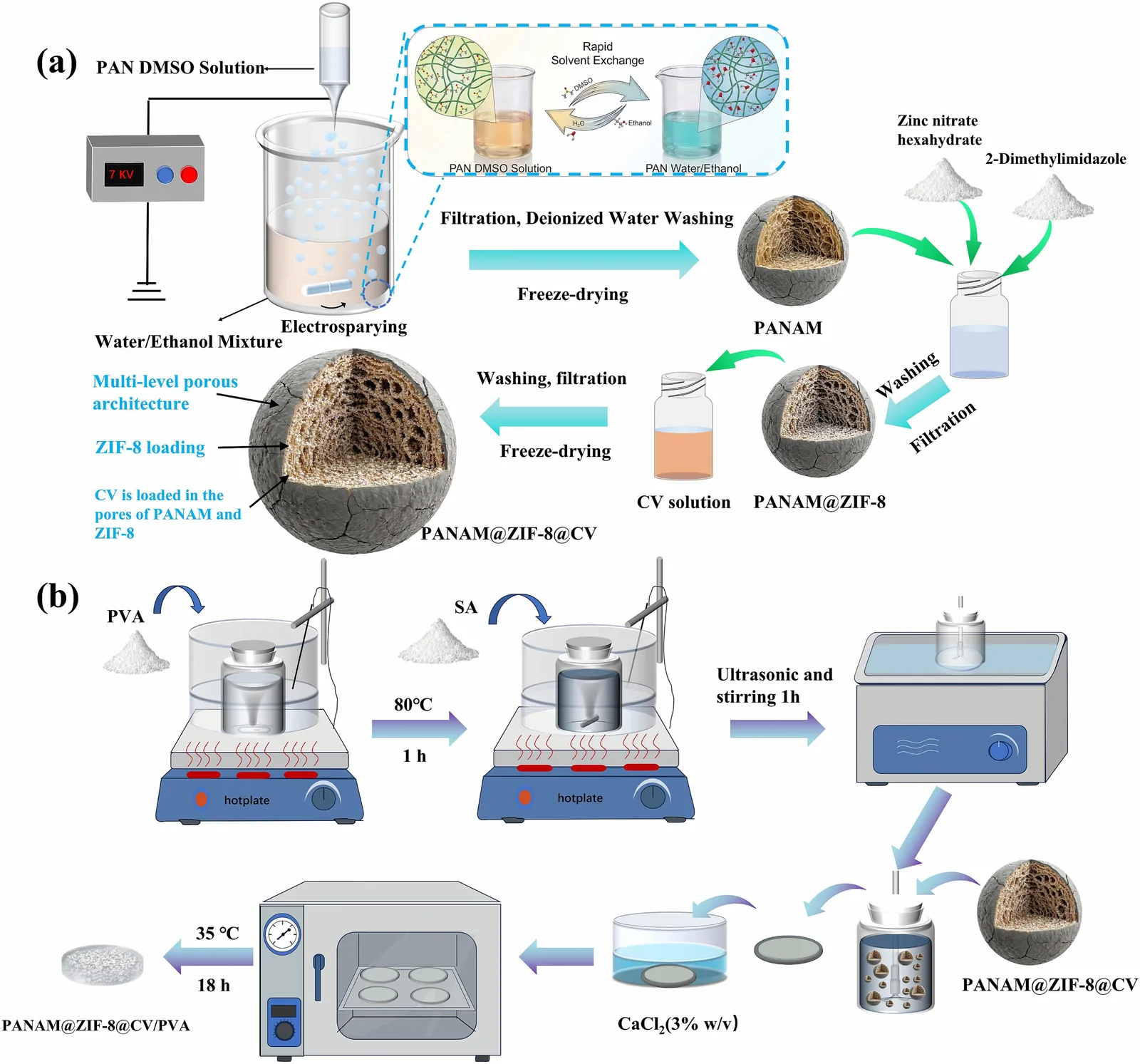
(a) Fabrication of PANAM@ZIF-8@CV by electrospinning coupled with in situ growth and immersion method; (b) Formation of the PANAM@ZIF-8@CV/PVA composite pad.

ZIF-8 crystals were synthesized by a facile liquid-phase precipitation method. Briefly, 1.17 g zinc nitrate hexahydrate (Zn(NO_3_)_2_·6H_2_O) was dissolved into 10 mL deionized water, whereas 22.70 g 2-methylimidazole was blended alone with 80 mL deionized water to yield a uniform aqueous solution. The zinc nitrate solution was quickly added into the vigorously stirred 2-methylimidazole solution, and a milky white suspension was formed instantaneously. After 15 min of gentle stirring, the suspension was centrifuged in two sequential stages at 5366 ×*g* for 15 min and 8385 ×g for 18 min, respectively. The recovered solid was rinsed three times with deionized water and purified by centrifugation. Ultimately, the washed product was subjected to vacuum freeze-drying for 48 h to yield pure ZIF-8 crystal powder.

ZIF-8@CV (carvacrol-loaded ZIF-8) was fabricated via a facile impregnation method. Briefly, 0.4 g of as-synthesized ZIF-8 powder was dispersed in 20 mL of methanol, and the suspension was ultrasonicated for 5 min to achieve homogeneous distribution. After complete dispersion, an equal volume of CV solution was dropwise added to the above mixture. The resulting system was magnetically stirred at room temperature for 48 h to facilitate CV immobilization and then kept static for 24 h to strengthen adsorption stability. The obtained precipitate was centrifuged at 12074 ×*g*, thoroughly washed with anhydrous methanol to eliminate free CV, and vacuum freeze-dried for 24 h to yield pure ZIF-8@CV composite powder.

### 2.3 Preparation of different composite pads

First, 1.6 g PVA was added into 20 mL of deionized water. The mixed solution was then kept under magnetic stirring at 80 °C with 500 rpm for one hour. Then, 0.2 g of sodium alginate (SA) was supplemented, and continuous stirring was conducted for another 1 h. The obtained mixture was ultrasonicated for 1 h to prepare a stable PVA/SA blended solution. Afterwards, 0.324 g PANAM@ZIF-8@CV was added under mild stirring to ensure its uniform dispersion in the PVA/SA matrix. The homogeneous mixed dispersion was divided into five equal aliquots as technical replicates, and each aliquot was evenly cast onto a circular polystyrene (PS) plate with a diameter of 3.5 cm. The plate was soaked in 3% (*w*/*v*) calcium chloride solution for 5 min to induce ionic crosslinking. Thorough washing with deionized water was performed to clear away residual impurities and unreacted materials, after which the sample was vacuum-dried at 35 °C for 18 h. The resulting PANAM@ZIF-8@CV/PVA composite pad was easily peeled off the PS template and preserved in a sealed desiccator (50% RH) for further experiments. The detailed preparation process is illustrated in Fig. 1b.

To further evaluate the comprehensive performance, different fillers were separately incorporated into the PVA/SA matrix following the above procedure to obtain a series of composite pads (PANAM@CV/PVA, ZIF-8@CV/PVA and CV/PVA) with identical CV content. Meanwhile, blank PVA/SA pad without CV was fabricated and denoted as PVA for comparative analysis. The detailed dosages of each filler are summarized in Table S2.

### 2.4 Characterization of different fillers and composite pads

X-ray diffraction analysis (XRD, D8 Advance, Germany) was employed to investigate the crystalline structure of various samples. A Thermo Scientific K-Alpha X-ray photoelectron spectrometer (Thermo Fisher Scientific, USA) equipped with a monochromatic Al Kα source was used to investigate the chemical states of the fillers. Wide-scan and high-resolution narrow scans (Zn, C, N) were collected and further analyzed using CasaXPS software. Nitrogen adsorption–desorption tests were performed on a Micromeritics ASAP 2460 analyzer (Micromeritics Instrument Corporation, Georgia, USA) to acquire the BET specific surface area values. The NLDFT model was also employed to calculate the pore volume as well as pore size distribution.

FESEM (Nova NanoSEM 450, FEI, USA) was utilized to observe the surface morphologies of various fillers and composite pads, along with the cryo-fractured cross-sections of the samples. All composites were fractured in liquid nitrogen to expose complete cross-sectional microstructures. EDS (Ultim Max, Oxford Instruments, UK) was further applied to analyze the elemental distribution and contents on the samples from FESEM micrographs.

ATR-FTIR spectra of diverse composite samples were acquired on a Fourier transform infrared spectrometer (Spectrum 3, PerkinElmer, USA) in the range of 4000–400 cm^−1^. Thermogravimetric properties were investigated via TGA and DTG using a Netzsch STA 449 F3 simultaneous thermal analyzer (Germany). Under nitrogen flow conditions, all samples were subjected to heat treatment from room temperature up to 800 °C with a ramp rate of 10 °C/min. A UV-2600i spectrophotometer (Shimadzu, Japan) was utilized to test the light transmittance of diverse composite pads at wavelengths ranging from 200 to 800 nm.

### 2.5 Sustained release performance of different pads

To assess the sustained-release behavior of the as-prepared composite pads, a set of carvacrol standard solutions with gradient concentrations (0.02, 0.04, 0.06, 0.08, 0.10 mg/mL) were prepared. Anhydrous ethanol was used as the blank, and the absorbance of each standard solution was determined at 275 nm. The corresponding standard curve was fitted with the regression equation: y = 36.91*x + 0.0554 (R^2^ = 0.998).

For the sustained release test, 0.5 g of each composite sample was immersed in 20 mL of anhydrous ethanol and shaken at 25 °C and 100 rpm. At predetermined time intervals (0, 6, 12, 24, 48 h, and every 24 h thereafter until 240 h), 1 mL of the release medium was withdrawn, and 1 mL of anhydrous ethanol was immediately replenished to maintain a constant total volume. The absorbance of each sample solution was detected at 275 nm. The cumulative release amount of carvacrol was quantified based on the standard curve in accordance with Eq. (1):

(1) $$\mathrm{Cumulative}\;\text{release}\;\left(\%\right)=\frac{M_{t}}{M_{0}}\times 100\%$$

*M*_*t*_ in the Equation represents the cumulative amount released up to time *t* (mg); *M*_*0*_ denotes the initial total loading (mg) of CV in different composite pads, which is determined by extraction as described in Sections S1.2 and S2.1.

### 2.6 Water vapor permeability (WVP) of different composite pads

Gravimetric measurements were conducted to obtain the water vapor permeability (WVP) values for the composites. Briefly, anhydrous calcium chloride was added into 10 mL centrifuge tubes, and the tube openings were tightly sealed with the as-prepared composite pads. All samples were placed in a desiccator with distilled water at the bottom, which was then stored in a forced-air oven at 25 °C and 100% relative humidity (Ren et al., 2023). The samples were weighed daily for seven consecutive days. Finally, the WVP values (kg·m^−1^·s^−1^·Pa^−1^) were calculated according to Eq. (2):

(2) $$\mathrm{WVP}=\frac{\mathrm{Vm}\times d}{A\times t\times \mathrm{Vp}}$$

Among these, *∆m* represents the mass change of the test sample over a time interval, *d* denotes the pad thickness, *A* indicates the area of the moisture transmission cup through which water vapor passes, *t* signifies the weighing interval time, and *∆p* denotes the water vapor pressure difference across the composite pad.

### 2.7 Antioxidant properties of different composite pads

To conduct the DPPH radical scavenging evaluation, 1 mg DPPH powder was dispersed and dissolved in 50 mL absolute ethanol under ultrasonication. Subsequently, the solution was diluted to adjust its absorbance at 517 nm to the range of 0.6–1.0 by using a UV-2600i spectrophotometer (Shimadzu, Japan). All composite pads were equilibrated for 3 days, then dispersed in absolute ethanol to prepare sample solutions at a concentration of 1 mg/mL. Each sample solution was sealed in a centrifuge tube and treated separately: (1) ultrasonication for 20 min; (2) ultrasonication for 40 min followed by 2 min of ultrasonic cell grinding with circulating water cooling. Subsequently, 1 mL of sample solution was mixed with 1 mL of diluted DPPH solution and incubated in the dark for 30 min. The absorbance was measured at 517 nm and defined as *A*_*1*_. Two controls were set: *A*_*0*_ (1 mL ethanol +1 mL DPPH solution) and *A*_*2*_ (1 mL sample solution +1 mL ethanol). Eq. (3) was adopted to calculate the DPPH radical scavenging rate:

(3) $$\text{DPPH scavenging activity}\;\left(\%\right)=\left(1‐\frac{A_{1}‐A_{2}}{A_{0}}\right)\times 100\%$$

For the ABTS radical scavenging assay, equal volumes of 7.0 mmol/L ABTS and 2.45 mmol/L potassium persulfate solution were combined. The mixture was incubated at 4 °C in darkness for 16 h to complete the reaction and form blue-green ABTS^+^ radical cation stock solution. The stock solution was diluted with PBS buffer (pH 7.4) to attain an absorbance of 0.70 ± 0.05 at 734 nm, namely the ABTS^+^ working solution (Eze et al., 2024). Two batches of 1 mg/mL sample solutions for each group were prepared in the same manner as described above. An equal volume (1 mL) of sample solution was fully mixed with fresh ABTS^+^ working solution. After standing in darkness at room temperature for 6 min, the solution absorbance was recorded at 734 nm. The ABTS^+^ radical scavenging activity was calculated based on Eq. (4):

(4) $${\mathrm{ABTS}}^{+}\;\mathrm{scavenging}\;\mathrm{activity}\;\left(\%\right)=\left[1-\left(\frac{B_{1}‐B_{2}}{B_{0}}\right)\right]\times 100\%$$

As for the above parameters, *B*_*1*_ denotes the absorbance of the mixture of sample and ABTS^+^ working solution, *B*_*2*_ is the absorbance value without ABTS participation, while *B*_*0*_ is measured from the solution with no sample added.

### 2.8 Antibacterial performance of various PVA composite pads

The zone of inhibition assay was used to evaluate the antibacterial performance of composite pads. The strains of *Escherichia coli (E. coli)* and *Staphylococcus aureus (S. aureus)* were independently cultured in Luria-Bertani (LB) broth and incubated at 37 °C for 12 h in a constant-temperature shaking incubator to obtain bacterial cells in the logarithmic growth phase. Bacterial suspensions were adjusted to an absorbance of 0.2 at 600 nm with sterile normal saline to unify bacterial concentration. The bacterial suspension was evenly smeared onto plate count agar using a sterile cotton swab to generate a uniform bacterial lawn (Li et al., 2024).

Circular sample pads (6 mm in diameter), including PANAM@ZIF-8@CV/PVA, PANAM@CV/PVA, ZIF-8@CV/PVA, CV/PVA and blank PVA, were prepared and stored for 0, 5, and 30 days. All samples were gently placed flat on the bacterial-covered agar surface to avoid air bubbles and displacement. The plates were placed in a thermostatic incubator and incubated undisturbed at 37 °C for 18 h. After incubation, bacterial growth was observed, and the formation of clear inhibition zones around each sample was recorded. The morphology and diameter of inhibition zones were measured by a vernier caliper (precision: 0.01 mm). All data were summarized and statistically analyzed to compare the antibacterial differences among composite pads with different formulations.

### 2.9 Preservation study of blueberries

A batch of fresh blueberries was obtained from a local market. Fruits with uniform size and maturity were screened to ensure consistent experimental results in the preservation test. PS containers (7.5 × 7.5 × 4 cm) were used in this study, and nine blueberries were placed in each container. All containers were cleaned with deionized water and completely dried. The lids consisted of perforated DuPont paper, and four identical composite pads were fixed on the inner surface. Another layer of DuPont paper was covered and sealed to prevent moisture uptake by the pads, which could interfere with experimental results. Six sample groups were established, among which the blank group used pure PS boxes without composite pads. Each group included three parallel replicates. All samples were stored in a constant temperature and humidity chamber (HRY-250, Safu Instruments, China) at 25 °C and 60% relative humidity. Physicochemical indices of blueberries were measured at scheduled intervals (0, 3, 6, 9, 12, 15 days) to compare the fresh-keeping effects of different composite materials.

#### 2.9.1 Determination of weight loss rate

Weight loss rate of blueberries was calculated according to their mass alterations, which were measured by an analytical balance during storage. The initial weight of the blueberries on Day 0 was recorded as *W*_*0*._ Subsequently, at the same time intervals on days 3, 6, 9, 12, and 15, the blueberries' weight was accurately measured and recorded as *W*_*t*._ The weight loss rate was then calculated using the Eq. (5):

(5) $$\mathrm{Weight}\;\mathrm{loss}\;\mathrm{percentage}\;\left(\%\right)=\frac{W_{0}‐W_{t}}{W_{0}}\times 100\%$$

#### 2.9.2 Determination of firmness

Blueberry firmness at different storage intervals was determined by a texture analyzer (TA-XT Plus C, Stable Micro Systems, UK). The relevant parameters of the P36R probe were set as follows: pre-test speed of 2 mm/s, test speed of 1 mm/s, and post-test speed of 2 mm/s. Each fruit was tested at the equatorial position, and three parallel samples were measured for every treatment group.

#### 2.9.3 Determination of vitamin C (VC) content

The Vitamin C content in blueberries was determined by 2,6-dichloroindophenol titration in accordance with GB 5009.86–2016. After dilution with 20 g/L oxalic acid solution, the sample filtrate was titrated against calibrated 2,6-dichloroindophenol solution to an endpoint of lasting pink hue maintained for 15 s. A blank titration using pure oxalic acid solution was conducted in parallel. The vitamin C content (mg/100 g) was calculated by Eq. (6):

(6) $$X\;\left(\mathrm{mg}/100g\right)=\frac{\left(V‐V_{0}\right)\times T\times A}{m}\times 100\%$$

In this Equation, X is the L (+)-ascorbic acid content of the sample (mg/100 g); *V* and *V*_*0*_ are respectively the 2,6-dichloroindophenol consumption (mL) for sample and blank titration. *T* refers to the titre of the indicator solution (mg/mL), namely the ascorbic acid mass equivalent to 1 mL of the solution and it was calculated via Eq. (7). *A* is the sample dilution factor, and *m* is the sample weight (g). All results were recorded as the average of three parallel replicates.

(7) $$T\;\left(\mathrm{mg}/\mathrm{mL}\right)=\frac{c\times V}{V_{1}‐V_{0}}$$

Where, *c* is the mass concentration of ascorbic acid standard solution (mg/mL); *V* is the volume of pipetted standard solution (mL); *V₁* and *V₀* are the volumes of 2,6-dichloroindophenol solution consumed in standard and blank titrations, respectively (mL).

#### 2.9.4 Determination of total soluble solids (TSS) and titratable acidity (TA)

The total soluble solids (TSS) content of blueberries was determined using a handheld refractometer (ATAGO, PAL-α, Japan) and expressed as Brix (%). Briefly, blueberry samples were fully ground and then centrifuged to collect the supernatant, which was used for subsequent experimental analyses. A 100 μL aliquot of the blueberry juice was carefully placed onto the measurement area of the refractometer, and the TSS value was recorded. Each sample was measured in triplicate to ensure the reliability of the experimental data.

For titratable acidity (TA) determination, the obtained juice was diluted with distilled water. One drop of phenolphthalein indicator was added, and the mixture was titrated with 0.1 M standard sodium hydroxide solution until a faint pink color appeared and persisted for at least 30 s. The consumption volume of NaOH solution was recorded, and the TA content was calculated according to the Eq. (8):

(8) $$\mathrm{TA}\;\left(\%\right)=\frac{c\times V\times k\times F\times 100}{m}$$

In the above formula, *c* is the molarity of standard NaOH solution, *V* is the consumed titration volume of the solution in milliliters, *k* denotes the acid conversion constant (0.064 for citric acid), *F* is the dilution factor, and m represents the sample weight in grams.

#### 2.9.5 Respiratory intensity test

To determine the respiration rate of blueberries, the samples were placed in a sealed container, and the respiration rate was calculated by measuring the accumulated CO_2_ concentration over a predetermined period. The calculation equation was presented in Eq. (9):

(9) $$\text{Respiratory intensity}\;\left(\mathrm{mg}\;{\mathrm{CO}}_{2}\cdot {\mathrm{kg}}^{‐1}\cdot h^{‐1}\right)=\frac{\left(V_{1}-V_{2}\right)\times C\times 44}{G\times t}$$

In this equation, *V*_*1*_ indicates the oxalic acid volume consumed in blank titration (mL); *V*_*2*_ indicates the oxalic acid volume consumed in sample titration (mL); *C* refers to the molarity of H_2_C_2_O_4_ (mol/L); *G* stands for the mass of blueberry samples (kg); *t* represents the CO_2_ absorption duration (h) in a desiccator.

#### 2.9.6 Taste profile analysis of blueberries

The taste profile characteristics of blueberry juice (extracted and diluted) from samples stored for 0, 7 and 15 days were analyzed using an electronic tongue (SA402B Plus-EX, Insent, Japan). The determined taste indexes included sourness, bitterness, saltiness, umami, astringency, umami aftertaste, bitter aftertaste and astringent aftertaste. Each sample was tested in triplicate, and all results were visualized using sensory radar charts and principal component analysis (PCA). The PCA results were visualized as a biplot with 95% confidence ellipses for each treatment group, and permutational multivariate analysis of variance (PERMANOVA) was used to determine whether the differences were statistically significant.

### 2.10 Statistical analysis

All experimental data were presented as the mean ± standard deviation (SD). Statistical analyses were performed utilizing SPSS software (Version 27.0, SPSS Inc., Chicago, IL, USA). One-way analysis of variance (ANOVA) followed by Duncan's multiple range test was employed to assess the significance of differences between groups, and the value of *p* < 0.05 was regarded as statistically significant.

## 3 Results and discussion

### 3.1 The microscopic morphology of different fillers

FESEM was utilized to observe the microscopic morphologies of ZIF-8 and ZIF-8@CV (Fig. 2a, a′, b, b′). Pure ZIF-8 displayed a regular cubic structure with uniform dispersion, and the particle sizes distribution was largely centered at 150–300 nm. After CV encapsulation, the overall morphology and particle size of ZIF-8 showed no obvious change. Nevertheless, partial crystals presented irregular edges and fuzzy surfaces, which confirmed the effective incorporation of CV into the ZIF-8 structure (Li et al., 2023).

**Fig. 2:**
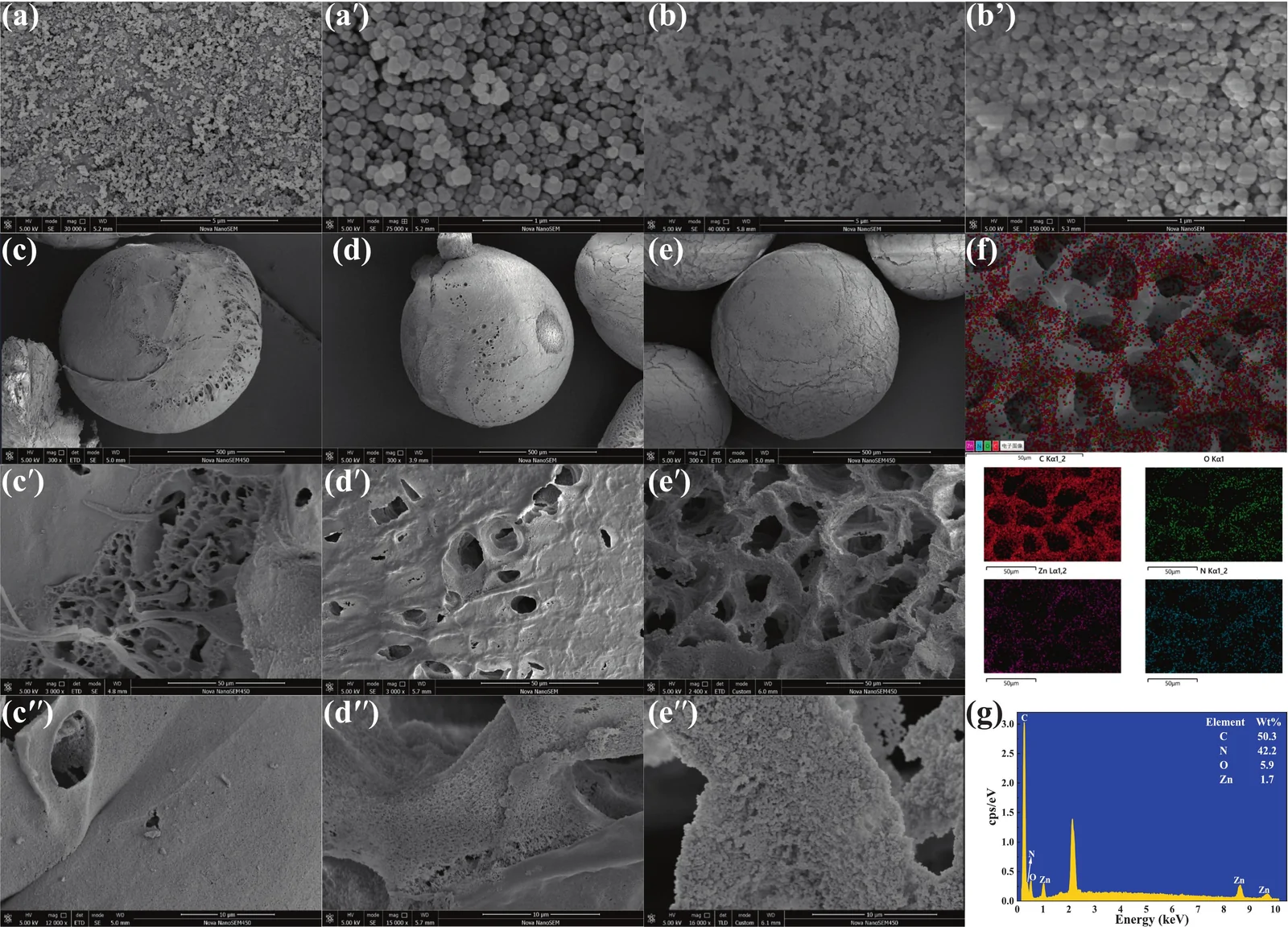
FESEM images of ZIF-8 (a, a′), ZIF-8@CV (b, b′), PANAM (c, c′, c″), PANAM@CV (d, d′, d″) and PANAM@ZIF-8@CV (e, e′, e″) at different magnifications. EDS mapping of PANAM@ZIF-8@CV for C, O, N, and Zn (f), and the corresponding elemental content distribution (g).

The micro-morphologies of pristine PANAM at different magnifications are shown in Fig. 2 (c, c′, c″). Pure PANAM exhibited a typical honeycomb-like architecture with diameters ranging from 400 to 500 μm. The microspheres possessed a smooth outer shell and an internal hierarchically porous structure, accompanied by a partial center-radiating structure. This unique structure provided abundant anchoring sites for reinforcing components (e.g., MOFs and CV) (Wang et al., 2024; Wang, Wang, et al., 2025), and laid a foundation for the preparation of PANAM@CV and PANAM@ZIF-8@CV hybrid fillers. Additionally, Fig. 2c″ showed tiny irregular particles attached to the PANAM surface, which were attributed to residual PAN monomers.

The FESEM images of CV-loaded PAN aerogel microspheres (PANAM@CV) are displayed in Fig. 2(d, d′, d″). The results demonstrated that the overall surface morphology of microspheres remained nearly unchanged after CV loading. At 15,000× magnification, the surface particles turned wrinkled and interconnected, which was probably caused by the surface wetting effect of CV. Meanwhile, FESEM images of PANAM@ZIF-8@CV are shown in Fig. 2(e, e′, e″). Typical and regular cubic crystals of ZIF-8 were clearly observed at 16000× magnification, verifying the successful decoration of ZIF-8 on PANAM microspheres, with no obvious difference in particle size.

The abundant micropores of ZIF-8, coupled with the hierarchical macro/mesopores of PANAM, jointly create a well-defined multi-level porous architecture. In contrast to the single-carrier system of pure ZIF-8 for direct CV loading, this distinctive structure restricts the diffusion of encapsulated CV inside microspheres and thus greatly extends the release period. The dual loading strategy endows the system with high CV encapsulation efficiency and achieves long-term steady release by virtue of porous diffusion resistance, PANAM shell shielding effect, and intermolecular weak interactions.

The EDS results of PANAM@ZIF-8@CV (Fig. 2f, g) revealed the presence of Zn and its uniform distribution on these aerogel microspheres, further verifying the successful preparation of PANAM@ZIF-8@CV.

### 3.2 Different properties of PANAM@ZIF-8@CV and other fillers

To study the loading situation of ZIF-8 and CV onto PANAM, XRD was conducted and the corresponding results are presented in Fig. 3a. The diffraction peaks of ZIF-8@CV and PANAM@ZIF-8@CV at approximately 7.30°, 10.30°, 12.60°, 14.75°, 16.40° and 17.95° correspond to the (110), (200), (211), (220), (310) and (222) crystal planes of ZIF-8, which are consistent with pure ZIF-8 (Li et al., 2023). This indicates that ZIF-8 was successfully loaded onto PANAM, and subsequent incorporation of CV barely altered the crystal structure of ZIF-8. Neat PANAM microspheres show a prominent broad diffraction peak at around 17°, assignable to the (100) crystal plane of the quasi-hexagonal polyacrylonitrile phase.

**Fig. 3:**
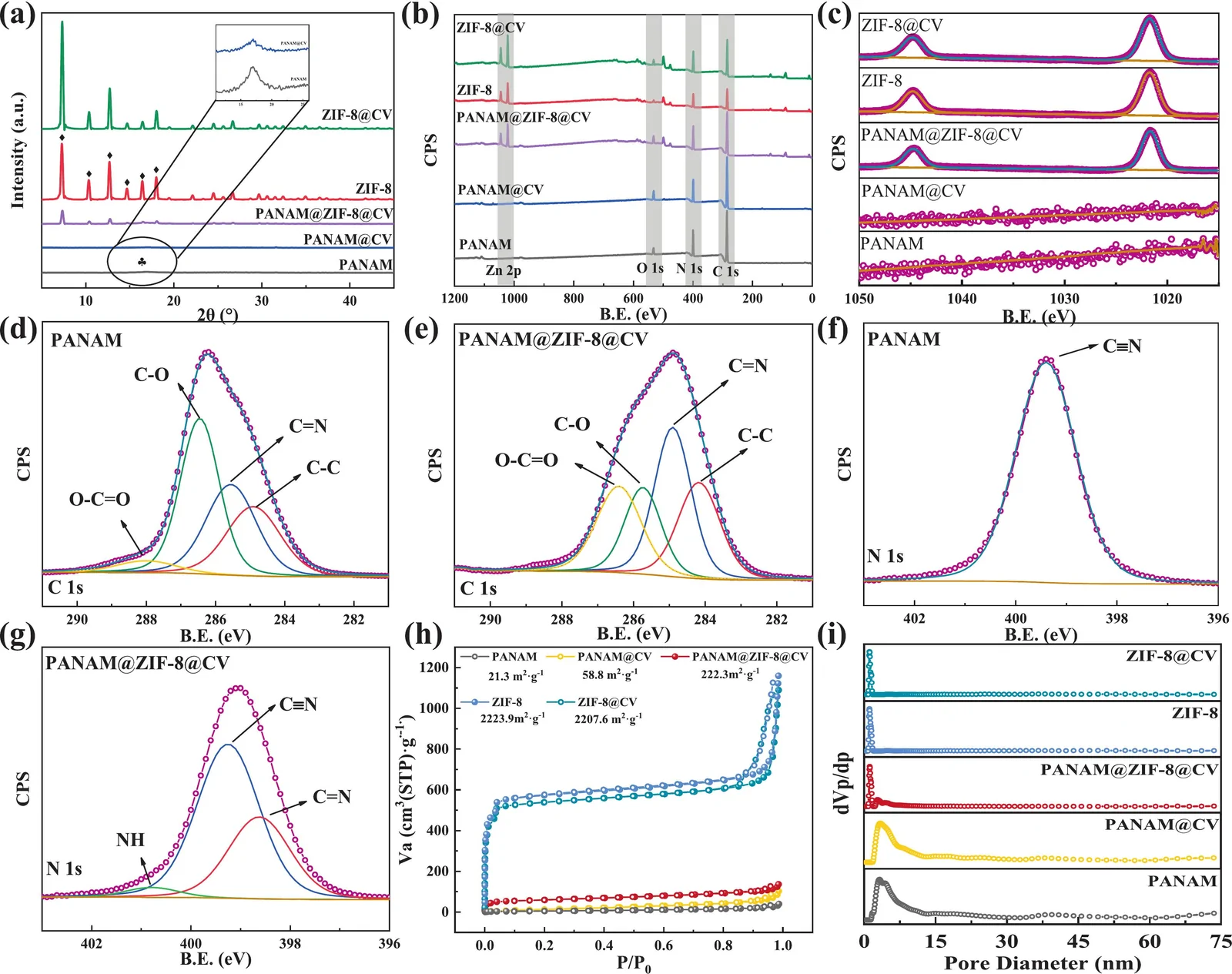
(a) XRD patterns, (b) full XPS spectra, and (c) Zn high-resolution narrow scan of PANAM, PANAM@CV, PANAM@ZIF-8@CV, ZIF-8, and ZIF-8@CV. High-resolution N narrow scans of PANAM (d) and PANAM@ZIF-8@CV (e), respectively; High-resolution C narrow scans of PANAM (f) and PANAM@ZIF-8@CV (g), respectively. (h) N_2_ adsorption-desorption isotherms of different fillers, and (i) pore size distributions derived from the NLDFT model.

The XPS results of PANAM, PANAM@CV, PANAM@ZIF-8@CV, ZIF-8, ZIF-8@CV are shown in Fig. 3b-g and Fig. S2. Narrow scans of C 1s, N 1s and Zn 2p were also performed to further clarify their chemical composition and valence state. No Zn 2p signal was detected for PANAM and PANAM@CV in Fig. 3b, c, which is in line with expectations. Distinct characteristic peaks were observed at ∼1021.5 eV and ∼1044.5 eV in other three samples, corresponding respectively to Zn 2p_3/2_ and Zn 2p_1/2_. The Zn 2p characteristic peaks of PANAM@ZIF-8@CV confirm that the ZIF-8 framework retains good structural integrity after the loading process.

All samples exhibited characteristic peaks assigned to C—C, C=N and C—O bonds (Fig. 3d, e and Fig. S2a–c); the incorporation of CV enhances the intensity of the O–C=O peak for ZIF-8@CV and PANAM@ZIF-8@CV. Given that the CV molecule itself does not contain the O–C=O functional group, this observation is primarily attributable to two main reasons. On the one hand, the phenolic hydroxyl groups of CV may form hydrogen bonds with the nitrogen atoms of the imidazole rings or defect-related sites in ZIF-8, thereby altering the local electron density and leading to shifts in the XPS signals. On the other hand, mild oxidation during material preparation induces slight oxidation of the side chains of CV, with a small number of carboxyl/carbonyl structures generated in situ. Consequently, the relative content of O–C=O functional groups increase, accompanied by a shift in peak position (Kerber et al., 1996; Turek & Stintzing, 2013).

The N 1 s spectra in Fig. 3f indicate that pure PANAM only presents the C≡N signal. In addition, the N 1s spectra of PANAM@ZIF-8@CV not only show the ZIF-8 characteristic C=N and NH peaks (Fig. 3g), but also display the C≡N peak derived from the PANAM skeleton. This further verifies the successful introduction and incorporation of ZIF-8 into the PANAM matrix.

To analyze the evolution of pore structure upon CV incorporation, additional N_2_ adsorption–desorption characterization was conducted. As shown in Fig. 3h, both ZIF-8 and ZIF-8@CV display Type I adsorption isotherms, indicating the presence of abundant micropores (< 2 nm) in the ZIF-8 framework, which can be further confirmed by the corresponding pore size distribution curves in Fig. 3i. Loading CV molecules only slightly occupies the micropores of ZIF-8 without destroying its porous framework. The specific surface area of pure PANAM is approximately 21.3 m^2^·g^−1^, revealing its low porosity. By contrast, pristine ZIF-8 possesses a high specific surface area of 2223.9 m^2^·g^−1^. After the introduction of PANAM to fabricate PANAM@ZIF-8@CV, the specific surface area and pore volume of the composite decrease sharply, as illustrated in Fig. 3h. This phenomenon reveals that PANAM effectively blocks the pores of ZIF-8, creating a good sealing effect and imparting excellent sustained-release properties to the material. Such a structural design is rationally optimized, where the outer PANAM layer can effectively avoid the premature leakage of internal CV molecules.

### 3.3 Morphology, element distribution and functional groups of different composite pads

Surface and cross-sectional morphologies of the PANAM@ZIF-8@CV/PVA composite at various magnifications are presented in Fig. 4(a, a′, a″) (surface morphology) and Fig. 4(d, d′, d″) (cross-sectional morphology), respectively. Obvious protrusive structures can be observed on the composite surface, which originate from the dispersion of PANAM within the PVA matrix. Apart from a small number of partially exposed particles, the fillers exhibit excellent interfacial compatibility with the polymer matrix. The cross-sectional morphology reveals that numerous PAN microspheres with a diameter of approximately 500 μm are uniformly and tightly embedded in the PVA matrix. This indicates that the ionically crosslinked network constructed by SA and calcium ions in the PVA matrix can effectively encapsulate and immobilize PANAM, thereby achieving a dual controlled-release effect benefiting from the synergism of the polymer matrix and microspheres. Meanwhile, Fig.4d″ further confirms that ZIF-8 is successfully immobilized on the surface of cross-sectional PANAM. Collectively, these results verify the successful encapsulation of PANAM@ZIF-8@CV in the PVA matrix. This well-designed structure not only takes full advantage of the high CV loading capacity of PANAM and ZIF-8 to confine CV inside their internal pores and form tortuous diffusion pathways via the hierarchical porous network of PANAM and ZIF-8, but also utilizes the barrier effect of the PANAM outer layer and PVA encapsulation to regulate the diffusion rate, thus realizing stable and long-term sustained release of CV.

**Fig. 4:**
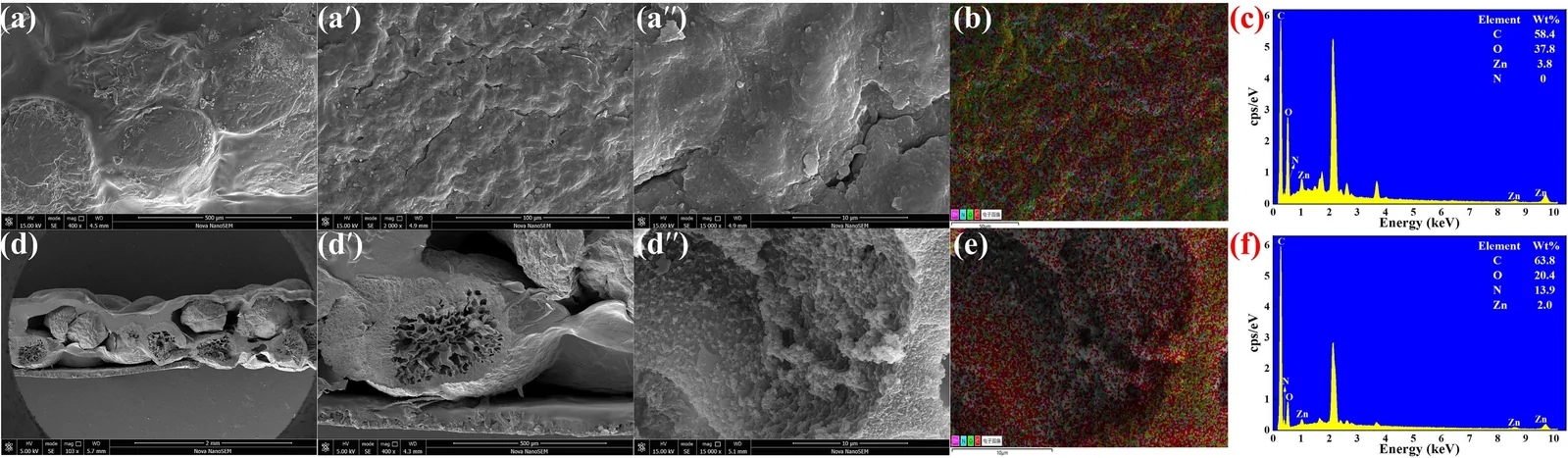
Surface FESEM images (a, a′, a″) and cryo-fractured cross-sectional FESEM images (d, d′, d″) of PANAM@ZIF-8@CV/PVA at different magnifications. EDS elemental mapping of PANAM@ZIF-8@CV for C, O, N and Zn (b), and the corresponding elemental distribution (c). EDS elemental mapping of the cryo-fractured cross-section for C, O, N and Zn (e), and the corresponding elemental distribution (f).

The EDS spectra and elemental content distribution corresponding to Fig. 4a′ and Fig. 4d″ are shown in Fig. 4b, c and Fig. 4e, f, respectively. Surface EDS results of the PANAM@ZIF-8@CV/PVA composite show no detectable nitrogen (N) signal, implying the absence of residual acrylonitrile. This confirms that PANAM@ZIF-8@CV is well encapsulated by the PVA matrix, which effectively inhibits the leaching of acrylonitrile monomers. Meanwhile, cross-sectional EDS elemental analysis confirms even dispersion of C, O, N and Zn in the composite structure, attesting to the well-constructed PANAM@ZIF-8@CV/PVA composite pad.

The ATR-FTIR results of different composite pads are presented in Fig. 5a. In pure PVA, the characteristic absorption peak at 3363 cm^−1^ is attributed to O—H stretching vibrations, whereas the band at 2955 cm^−1^ arises from saturated C—H stretching vibrations. The band at 1086 cm^−1^ is mainly attributed to the C—O stretching vibration of the PVA molecular chain, and the peak at 1298 cm^−1^ arises from the O—H in-plane bending vibration in the PVA backbone. These four absorption bands constitute the typical characteristic FTIR peaks of PVA. In addition to the above peaks, two obvious characteristic absorption peaks appear at 1632 cm^−1^ and 1420 cm^−1^ which can be ascribed to the asymmetric and symmetric stretching of alginate carboxylate groups (–COO^−^) in sequence (Bialik-Wąs et al., 2021; Daemi & Barikani, 2012).

**Fig. 5:**
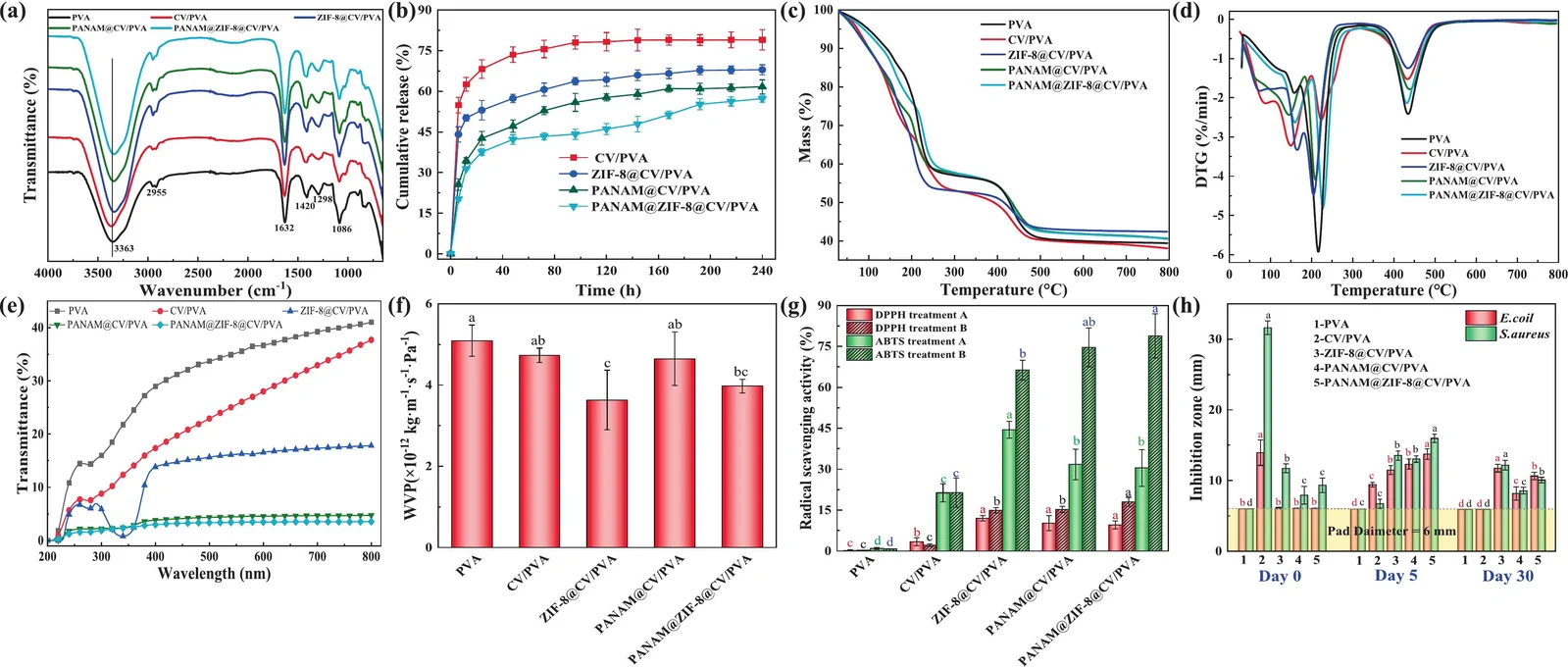
(a) ATR-FTIR spectra of different composite pads; (b) Cumulative release behavior of CV from different composite pads within 10 days; (c) TGA curves, (d) DTG curves, (e) UV–Vis light transmittance curves, and (f) Water vapor permeability of different composite pads; (g) DPPH and ABTS radical scavenging activities of different composite pads (Treatment A: ultrasound treatment for 20 min; Treatment B: ultrasound treatment for 40 min followed by ultrasonic cell grinding for 2 min); (h) Inhibition zone diameters against *E. coli* and *S. aureus* of different composite pads after different storage days. Statistical significance was analyzed using one-way ANOVA followed by Duncan's multiple range test. Different letters of the same color above columns of the same type, under the same treatment or measured on the same days, indicate significant differences (*p* < 0.05).

After incorporating different fillers (PANAM, ZIF-8, and CV), no significant changes were observed in the FTIR absorption peaks of the composite pads, indicating that these fillers were well encapsulated in the polymer matrix. Moreover, compared with the pure PVA sample, the O—H stretching peaks at 3363 cm^−1^ for the composites (ZIF-8@CV/PVA, PANAM@ZIF-8@CV/PVA) shifted to lower wavenumbers, suggesting the formation of weak hydrogen bonds (e.g., O–H…C ≡ N and O–H…N) between the fillers (PANAM, ZIF-8) and the PVA matrix (Yang et al., 2020; Ye et al., 2019). Notably, no characteristic peaks appeared around 2244 cm^−1^, the main characteristic peak of pristine PANAM (Fig. S1a), confirming the absence of cyano groups (–C ≡ N) on the PVA surface in PANAM@ZIF-8@CV/PVA and PANAM@CV/PVA composites. Furthermore, the FTIR results of different fillers in Fig. S1a show that CV can form hydrogen bonds with PANAM and ZIF-8.

### 3.4 Releasing behavior of CV in different composite pads

The release behavior of CV was evaluated by quantifying the cumulative release amount of CV from various composite pads which can be seen in Fig. 5b. The pure CV/PVA sample exhibited a significant burst release within the initial 12 h, with 62.6% of CV released. The release profile gradually leveled off after 96 h. This behavior is mainly attributed to the high hydrophilicity of PVA, which is easily swollen by absolute ethanol. Meanwhile, the pad lacks effective binding sites to constrain CV, leading to the rapid diffusion and outflow of CV in ethanol. In addition, the gradual relaxation of the pad structure during long-term release causes the volatilization and loss of most active substances (Min et al., 2024).

ZIF-8@CV/PVA, PANAM@CV/PVA and PANAM@ZIF-8@CV/PVA also showed an initial burst release within 12 h, indicating that prolonged shaking could release the loosely adhered CV on the surface or inside the composite pads. Unlike that of CV/PVA, the cumulative release amount of CV for the three composite materials continued to increase from 96 h to 240 h. These results demonstrate that PANAM and ZIF-8 in the composite system can effectively restrict CV via hydrogen bonding and realize its slow sustained release. In particular, PANAM@ZIF-8@CV/PVA presented a secondary burst release at 192 h, suggesting an excellent synergistic inhibition and controlled-release effect between PANAM microspheres and ZIF-8. PANAM microspheres improve the compactness of the pad structure and reduce the ethanol-induced swelling. CV is encapsulated in the porous channels and loading sites of PANAM and ZIF-8, which reduces its non-specific diffusion. Moreover, ZIF-8 can stably adsorb CV through its high specific surface area and coordination interaction, further delaying the migration of CV molecules (Angeli et al., 2025). Long-term oscillation at 100 rpm destroyed the microsphere composite structure and released the stably adsorbed CV, thus resulting in the secondary burst release at the later stage.

Kinetic modelling was performed with four release models: zero-order, first-order, Higuchi, and Ritger–Peppas, where the Ritger–Peppas model was restricted to datasets with cumulative release ≤60%. As summarized in Table S4, the fitting results demonstrate significant differences in the release behaviors of the four CV sustained-release systems. The zero-order model shows poor fitting performance, the first-order model yields moderate fitting, the Higuchi model provides the best fit for the entire release period, and the Ritger–Peppas model (fitted to the first 60% release data) enables accurate determination of the release mechanism. All systems exhibit n values below 0.45, indicating that the release process is dominated by Fickian diffusion (Tong et al., 2026). The release rate decreases gradually with the increasing complexity of the carrier structure, following the order of CV/PVA > ZIF-8@CV > PANAM@CV/PVA > PANAM@ZIF-8@CV/PVA. Specifically, in the late release stage, as CV encapsulated in the outer layer of various composite materials is gradually released, the amount of tightly entrapped residual CV decreases continuously, leading to a plateaued release trend observed in the release profiles. In contrast, this plateauing tendency emerges much later in the PANAM@ZIF-8@CV/PVA system, indicating that PANAM@ZIF-8 can effectively immobilize more CV within its core layer. This structural characteristic enables steady and continuous CV release in the early stage, reflecting its advantage in long-term sustained release. Notably, the cumulative release of CV from this composite remains below 60% over a 240-h release period, demonstrating favorable sustained-release performance and certain application potential relative to some previously reported systems. (Nong et al., 2025; Wang, Zhou, et al., 2025a). The synergistic interplay between PANAM and ZIF-8 within the PANAM@ZIF-8@CV/PVA composite yields the slowest CV release and optimal controlled-release performance, effectively mitigating initial burst release and sustaining long-term stable delivery. Collectively, this dual-loading system endows the composite with exceptional long-term sustained-release behavior and superior capability for CV encapsulation and controlled release.

### 3.5 Thermal and barrier properties of different GL composite pads

The TGA and DTG curves of various PVA composite pads were used to evaluate their thermal properties, as presented in Fig. 5c, d. The first weight-loss stage of all composite materials occurs below 200 °C. All PVA-based samples exhibit initial mass loss upon heating, which is attributed to the volatilization of adsorbed and bound water within the polymer network, as well as the rapid evaporation of CV. In this stage, neat PVA shows the lowest weight-loss rate, corresponding to the weakest peak in the DTG profiles. This is because the sodium alginate and calcium ions in pure PVA can better retain bound water, with no additional mass loss from CV volatilization. PANAM@ZIF-8@CV/PVA also exhibits relatively low mass loss at this stage, since CV is confined within the microspheres and ZIF-8 frameworks and thus less prone to volatilization.

The second weight-loss stage ranges from 200 °C to 400 °C. In this interval, the hydroxyl side chains of the PVA matrix undergo decomposition, accompanied by decarboxylation and initial cleavage of glycosidic bonds in sodium alginate. Meanwhile, thermal degradation of the polyacrylonitrile main chains also takes place in PANAM@ZIF-8@CV/PVA and PANAM@CV/PVA (Zhang, Ma, et al., 2019). Neat PVA undergoes rapid decomposition in this stage with the maximum weight-loss rate, matching the strongest peak in the DTG curves. The DTG peaks (corresponding to the maximum mass loss rate) of PANAM@ZIF-8@CV/PVA in the above two stages shift toward higher temperatures compared with other composites, confirming favorable interfacial interactions between the filler and PVA matrix. Temperatures above 400 °C are assigned to the third weight-loss stage, which involves extensive main-chain scission and carbonization of PVA, as well as thorough decomposition of the calcium alginate polysaccharide backbone. This process leads to considerable mass loss, as also reflected by the distinct DTG peaks (Yang et al., 2014; Zhang, Han, et al., 2019). Finally, ZIF-8@CV/PVA presents the highest char residue, while the CV/PVA composite displays the lowest residual mass. This is because ZIF-8 exhibits the highest char residue (Fig. S1b), while PANAM disrupts the tight interchain interactions within the PVA matrix, leading to more facile mass loss of the matrix.

The UV–visible light and water vapor barrier properties of different composite materials are presented in Fig. 5e and Fig. 5f. The results indicate that the incorporation of various fillers can effectively improve the UV–visible light barrier performance of the composites. Among all groups, ZIF-8@CV/PVA and CV/PVA exhibit enhanced UV and visible light barrier capacities to a certain extent. On the one hand, ZIF-8 dispersed in the matrix can induce efficient light scattering and multiple light reflection. On the other hand, CV molecules contain aromatic rings and conjugated structures, which endow the material with moderate light absorption capacity. Notably, an abnormal reduction in transmittance of ZIF-8@CV/PVA was observed near 350 nm. This phenomenon may be attributed to two factors: the aromatic structure and phenolic hydroxyl groups of CV strengthen the absorption in the near-ultraviolet region, and the agglomeration of ZIF-8 particles further enhances the light scattering effect at this specific wavelength. In comparison, PANAM@ZIF-8@CV achieves the optimal light barrier performance. The 500 μm-scale PANAM microspheres increase the thickness of the PVA composite pad and effectively block and reflect most UV and visible light. In addition, functional groups such as cyano groups in the molecular structure of PAN possess intrinsic UV absorption ability. Meanwhile, the uniform loading and dispersion of ZIF-8 and CV efficiently fill the micropores inside the matrix, thereby further restricting the penetration of UV and visible light.

In terms of water vapor barrier performance, CV, as a small-molecule active substance, can fill the gaps of the polymer network and improve the compactness of composites. Hydrogen bonds can be established between the phenolic hydroxyls of CV and the hydroxyl groups in PVA/SA, which reduces the hydrophilicity of the pads and inhibits the adsorption and penetration of water molecules (Ning et al., 2022; Wang, Zhou, et al., 2025b). As porous nanoparticles, ZIF-8 can construct a physical barrier in the matrix to extend the diffusion path of water molecules. Meanwhile, its rigid structure restricts the movement of polymer chain segments, reduces the free volume, and lowers the water vapor permeability coefficient (Li et al., 2023). Among all the prepared composites, ZIF-8@CV/PVA presented the lowest water vapor permeability value and the optimal water vapor barrier property (*p* < 0.05). For PANAM@ZIF-8@CV/PVA, the introduction of large-size PANAM microspheres increased the thickness of composite pads, thereby forming an effective physical barrier and prolonging the diffusion path of water vapor. Nevertheless, the large microsphere size inevitably produced interfacial voids, which acted as preferential diffusion channels for water vapor and caused adverse effects on barrier performance. Fortunately, the microspheres were uniformly dispersed within the polymer matrix. In addition, sodium alginate and calcium ions formed a stable ionic crosslinking network in the PVA matrix, and hydrogen bonding was also generated between the matrix and fillers. These interactions effectively weakened the negative effect of interfacial voids. Accordingly, PANAM@ZIF-8@CV/PVA still exhibited the second-best water vapor barrier capacity.

### 3.6 Antioxidant properties of different composite pads

DPPH and ABTS free radical scavenging assays were used to evaluate the antioxidant properties of different composite pads under various treatment conditions, and the results are shown in Fig. 5g. The DPPH and ABTS free radical scavenging rates of pure PVA composite pads were nearly 0 under the two different treatment conditions (20 min ultrasound, 40 min ultrasound and 2 min ultrasonic cell grinding), exhibiting almost no antioxidant activity. Consistent with prior research, this phenomenon may occur because the molecular frameworks of PVA and SA lack hydrogen-donating and electron-donating active groups, limiting efficient free radical scavenging. (Tan et al., 2025; Younis et al., 2025). After the addition of CV, the DPPH and ABTS scavenging rates of the CV/PVA composite pad reached a maximum of 3.43% and 21.32%, respectively, showing a significant increase in antioxidant performance (*p* < 0.05). This is because CV, as a natural phenolic monoterpene compound, can donate hydrogen atoms through the phenolic hydroxyl groups in its molecules and then combine with free radicals to form stable structures, thereby exerting antioxidant effects (Karabagias et al., 2025). However, the CV loaded in this composite pad is prone to volatilization, resulting in low CV content and limited antioxidant performance.

Furthermore, the antioxidant properties of composite materials loaded with CV via fillers (ZIF-8@CV/PVA, PANAM@CV/PVA, PANAM@ZIF-8@CV/PVA) were improved to a certain extent. Under 20 min ultrasound treatment, ZIF-8@CV/PVA exhibited the highest DPPH and ABTS scavenging rates, which were 12.06% and 44.43%, respectively, and both were significantly higher than those of other composite pads (*p* < 0.05). This is because CV can be stably anchored in the porous metal-organic framework of ZIF-8 through hydrogen bonding and Zn^2+^ coordination and can be stably released under 20 min ultrasound treatment, thus exerting a certain antioxidant effect. In contrast, the CV in PANAM@ZIF-8@CV/PVA was better encapsulated in the dual system of PAN microspheres and ZIF-8, and the 20 min ultrasound treatment could not ensure the complete release of CV inside, thereby reducing the effectiveness of its antioxidant capacity. However, this also better illustrates the superior encapsulation effect of this dual-layer loading system on CV, which can also effectively prolong its sustained release. After 40 min ultrasound treatment combined with 2 min ultrasonic cell grinding process, PANAM@ZIF-8@CV/PVA exhibited the highest DPPH and ABTS scavenging rates, which were 18.06% and 78.85%, respectively, both higher than those of other groups (*p* < 0.05). This is attributed to the fact that the porous structure of ZIF-8 and the high specific surface area of polyacrylonitrile microspheres can better preserve CV; after further ultrasound and ultrasonic disruption treatment, more stored CV can be released, improving the contact efficiency between CV and free radicals, thereby significantly enhancing the antioxidant performance of the composite material (Li et al., 2025; Yu et al., 2021).

Notably, the ABTS radical scavenging activity of different composite pads was higher than their DPPH radical scavenging activity. This may be due to the smaller steric hindrance and easier electron transfer of ABTS^+^·, while DPPH· has a stable structure and larger steric hindrance, resulting in relatively low reaction efficiency between CV released from different composite pads and DPPH·.

### 3.7 Antibacterial properties of different composite pads

For composite pads utilized in food packaging, favorable antibacterial properties are indispensable. This study evaluated the antibacterial effects of different composite pads on *E. coli* and *S. aureus* after storage for 0, 5, and 30 days. The macroscopic antibacterial images and inhibition zone diameters are shown in Fig. 6 and Fig. 5h. The results reveal that the pure PVA pad shows negligible antibacterial activity against *E. coli* and *S. aureus*, with no distinct inhibition zone formed. However, the CV/PVA pad shows favorable antibacterial effects on both bacteria on day 0, with inhibition zone diameters enhanced to 13.93 mm and 31.60 mm compared with those of pure PVA (*p* < 0.05), respectively. This indicates that CV directly loaded in the PVA pad undergoes burst release, and the released CV suppresses ATPase activity, collapses the proton motive force (PMF) of the two bacteria, damages the structural integrity of microbial cell membranes, and triggers cytoplasmic leakage and oxidative damage, thereby exerting antibacterial effects (Gill & Holley, 2006; Khan et al., 2017). On Day 0, the three composite pads (ZIF-8@CV/PVA, PANAM@CV/PVA, and PANAM@ZIF-8@CV/PVA) exhibited weaker antibacterial activity against the two tested bacteria than CV/PVA, as evidenced by their smaller inhibition zone diameters (*p* < 0.05). This phenomenon indicates that less CV and Zn^2+^ were released from the three modified samples in the initial stage.

**Fig. 6:**
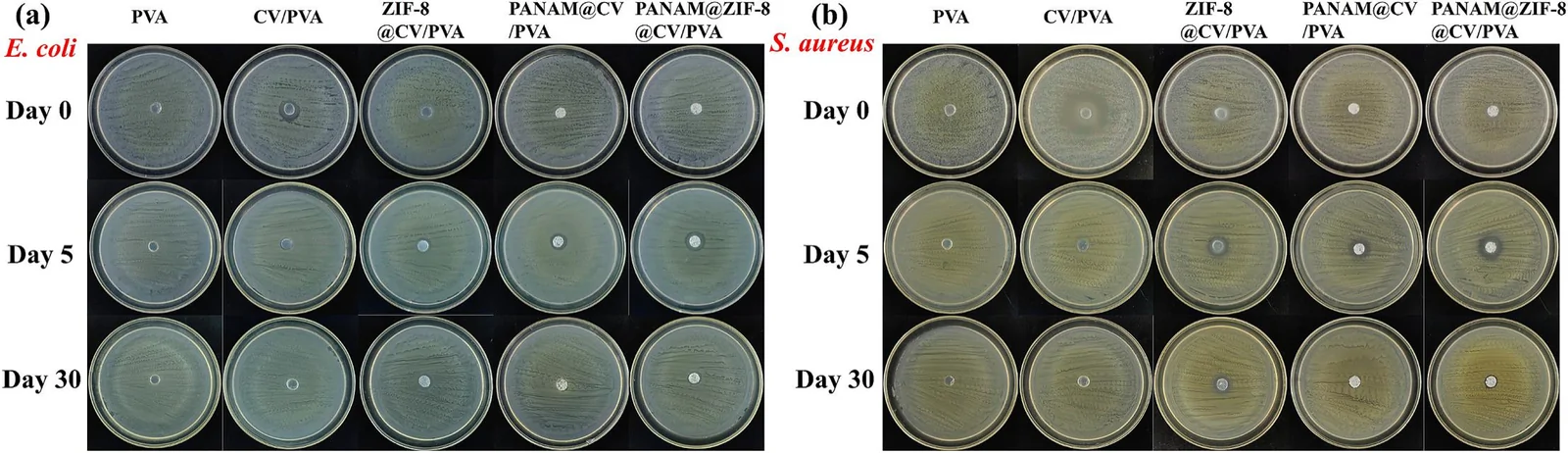
Photographs of antibacterial activities of PVA, CV/PVA, ZIF-8@CV/PVA, PANAM@CV/PVA, and PANAM@ZIF-8@CV/PVA against *E. coli* (a) and *S. aureus* (b) on Day 0, Day 5, and Day 30.

By Day 5, the inhibition zones of the CV/PVA composite pad against *E. coli* and *S. aureus* decreased significantly, indicating that a large proportion of CV in this composite pad had volatilized. In comparison, ZIF-8@CV/PVA, PANAM@CV/PVA, and PANAM@ZIF-8@CV/PVA all retain certain antibacterial properties. Notably, the PANAM@ZIF-8@CV/PVA demonstrated the optimal antibacterial performance compared with other groups (*p* < 0.05), with inhibition zone diameters of 13.77 mm against *E. coli* and 15.99 mm against *S. aureus*. This proves that the PANAM@ZIF-8@CV/PVA composite pad enables the uniform and long-term release of CV, achieving excellent antibacterial effects on both bacteria.

By Day 30, ZIF-8@CV/PVA, PANAM@CV/PVA and PANAM@ZIF-8@CV/PVA still retained measurable antibacterial activity, whereas the pristine CV/PVA sample exhibited no detectable antibacterial efficacy. This observation aligns with expectations: the incorporation of ZIF-8 and PANAM creates a diffusion barrier that effectively mitigates the burst release of CV typical of pristine CV/PVA pads, and this moderated initial release rate serves as a critical prerequisite for long-term sustained performance. Especially, ZIF-8@CV/PVA showed superior antibacterial activity to the other groups (*p* < 0.05), which was attributed to the sustained release of Zn^2+^ from the composite pad during long-term storage (Qin et al., 2025). Zinc ions destroy the integrity of bacterial cell membranes, cause the leakage of intracellular substances (K^+^, DNA, proteins), interfere with bacterial metabolism and enzyme activity, and ultimately lead to bacterial death (Cai et al., 2021; Li et al., 2025). In addition, nanostructured ZIF-8 enables the generation of reactive oxygen species (ROS). These species trigger bacterial oxidative stress, damage cell membranes, DNA and proteins, and ultimately achieve an oxidative bactericidal effect. The PANAM@ZIF-8@CV/PVA group also retained considerable antibacterial activity, with the inhibition zone diameters against *E. coli* and *S. aureus* being 10.71 mm and 10.14 mm, respectively. Although the presence of microspheres impedes Zn^2+^ release, the dual-loading structure enables sustained and significant long-term release of CV even after 30 days of storage, thereby maintaining favorable long-term antibacterial activity against bacteria.

Notably, all composite pads show stronger inhibitory effects on *S. aureus* than on *E. coli*. This observation can be explained by the absence of an outer membrane structure in Gram-positive bacteria, which allows CV to penetrate the cell wall more easily. In general, phenolic compounds display more pronounced antibacterial effects on Gram-positive bacteria compared with Gram-negative ones (Khwaza & Aderibigbe, 2025).

### 3.8 Preservation study of blueberries

#### 3.8.1 Conventional freshness preservation analysis

Five composite sample groups and one blank control group were used to determine the visual appearance, weight loss rate, firmness, total soluble solids (TSS), titratable acidity (TA), vitamin C (VC) and respiration rate of blueberries at 3-day intervals, in order to evaluate their preservation effect on blueberries. Macrographs of blueberries stored in different groups (Fig. 7a) clearly demonstrate the performance differences among the composite pads. Both the control group and PVA group exhibited severe shriveling and extensive black spotting, which could be attributed to the high postharvest respiration rates, excessive water loss and microbial contamination of blueberries. In contrast, blueberries preserved with CV-containing pads showed significantly improved freshness and remarkably fewer decay spots compared with the control and PVA groups. This improvement arises from the ability of CV to disrupt the cell membrane structure of surface microorganisms and inhibit their cellular metabolism, thus maintaining the sensory quality of berries (Medina-Jaramillo et al., 2020; Pie-Amill et al., 2024). Notably, blueberries stored in the PANAM@ZIF-8@CV/PVA group maintained high surface gloss, without obvious shrinkage and with fewer browning spots during storage, showing the best preservation effect.

**Fig. 7:**
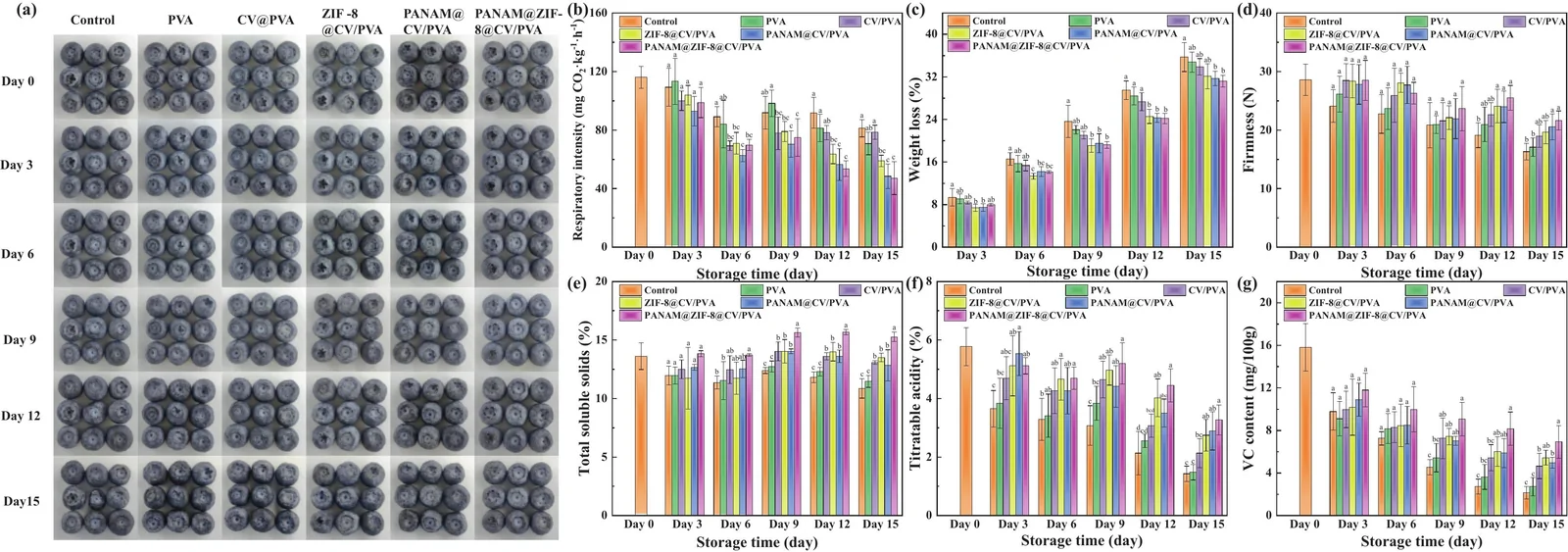
Preservation evaluation of blueberries with different sample groups at 25 °C and 60% RH. (a) Visual appearance photographs of blueberries; (b) Respiration intensity; (c) Weight loss rate; (d) Firmness; (e) TSS content; (f) TA content; (g) VC content of different groups during storage. Samples were measured every 3 days for a total storage period of 15 days. Statistical significance was analyzed using one-way ANOVA followed by Duncan's multiple range test. Different letters across columns on the same day indicate significant differences (*p* < 0.05).

Fig. 7b illustrates the variation in blueberry respiration rate during storage. According to the data, the respiration rate of blueberries on Day 0 was 115.65 mg CO_2_·kg^−1^·h^−1^. Throughout the storage period, the respiration rate generally showed a downward trend, but a temporary increase was observed on Day 9. This phenomenon could be attributed to extracellular secretions of grey mold, which caused the activities of antioxidant enzymes in blueberries to first increase and then decrease, leading to a stress-responsive respiratory peak (Jia et al., 2025; Liu et al., 2023). The PVA group partially reduced the respiration rate, with a value of 70.83 mg CO_2_·kg^−1^·h^−1^ and an inhibition rate of 12.90% compared with control group on Day 15. This reduction occurred because the composite pad restricted oxygen transfer to blueberry tissues, thus lowering respiration and respiratory metabolism. However, this pad lacked the ability to actively inhibit respiratory metabolism. In contrast, the incorporation of CV overcame this drawback and significantly decreased the respiration rate of the other four groups on Days 9 and 15. This effect was attributed to the antibacterial and antioxidant bioactivities of CV, whose components inhibited key respiratory metabolic enzymes and further suppressed respiration (Sun et al., 2021). Compared with the control group, the PANAM@ZIF-8@CV/PVA, PANAM@CV/PVA and ZIF-8@CV/PVA samples effectively reduced the respiratory rate of blueberries (*p* < 0.05). On Day 15, their respiratory inhibition rates relative to the control reached 41.96%, 40.33% and 27.43%, respectively. The PANAM@ZIF-8@CV/PVA pad showed the strongest inhibitory effect during the entire storage period. This excellent performance was ascribed to the controlled release of CV through the layered encapsulation system, which maintained an effective and safe concentration of CV inside the package to inhibit microbial growth and respiration (Zhang, Ma, et al., 2019).

Weight loss and firmness are critical indicators for evaluating the preservation performance of blueberries during storage. The results of weight loss and firmness are shown in Fig. 7c and Fig. 7d, respectively. Substantial water loss caused by transpiration and respiratory metabolism is the main reason for weight reduction in stored blueberries (Su et al., 2023). The untreated control group exhibited the highest weight loss rate, reaching 35.72% after 15 days. In contrast, composite materials incorporated with CV, especially the PANAM@ZIF-8@CV/PVA group, effectively reduced water loss. This was attributed to the hydrophobicity, water resistance and antibacterial activity of CV enabled by its long-term sustained release behavior. The treatment yielded an average weight loss rate of 31.20% after 15 days, which presented an obvious decrease compared with the control group (*p* < 0.05) (Bassey et al., 2025). For firmness, the control group showed the most significant decline, with a reduction of 42.74% within 15 days. This was mainly ascribed to pectin hydrolysis in the cell wall and loss of turgor pressure after harvest (Toledo-Guerrero et al., 2024; Wang, Wang, et al., 2025). Composite pads containing CV significantly inhibited blueberry softening by suppressing microbial growth, reducing the activities of softening-related enzymes, and delaying cell wall degradation (Medina-Jaramillo et al., 2020; Sun et al., 2021). Among all groups, the PANAM@ZIF-8@CV/PVA group maintained the highest firmness (21.64 N) after 15 days. This result originated from the dual barrier effect of PANAM microspheres and ZIF-8, which not only enhanced the controlled release of CV but also improved the physical barrier performance of the composite pad. The slow, sustained, and prolonged release of CV effectively delayed berry softening.

During blueberry storage, TSS, TA and VC levels are crucial markers reflecting flavor characteristics and nutritional properties, and their results are presented in Fig. 7e–g. As storage time progressed, both TSS and TA contents in blueberries declined. The control group exhibited a significant reduction in TSS by 19.9% and a sharp decrease in TA by 75.26% over 15 days. In contrast, the PANAM@ZIF-8@CV/PVA composite pads effectively inhibited the decrease in TSS content, which even slightly increased after 15 days. It also maintained a relatively high TA content, which was significantly higher than that of the control group (*p* < 0.05). This beneficial effect was attributed to the synergistic role of the composite pad in sustaining the effective release of CV, which effectively suppressed respiratory metabolism and retained moisture. This, in turn, helped preserve the sugar content in blueberries and effectively reduced organic acid consumption (Sun et al., 2025). As a critical nutritional indicator and antioxidant in blueberries, VC content reflects the freshness and quality deterioration of the fruit through its concentration changes (Adhikari et al., 2024). As shown in Fig. 7g, VC content in the control group underwent rapid oxidative degradation, with a retention rate of only 13.63% after 15 days. Composite pads containing CV (e.g., the PANAM@ZIF-8@CV/PVA and ZIF-8@CV/PVA groups) significantly preserved VC content in blueberries (*p* < 0.05). This was achieved through the potent antioxidant activity of CV, which scavenged reactive oxygen species (ROS) and inhibited ascorbate oxidase activity. Notably, blueberries in the PANAM@ZIF-8@CV/PVA group retained the highest VC content of 6.93 mg/100 g on Day 15. This outstanding performance was due to the synergistic effect between PANAM microspheres and ZIF-8, which ensured the long-term sustained release of CV and provided a robust barrier function. This minimized the consumption of VC and thus maximally preserved the nutritional quality of blueberries.

#### 3.8.2 Electronic tongue analysis and preservation mechanism

The taste profiles of blueberries under different preservation conditions were analyzed by using an electronic tongue on Day 0, 7, and 15, with the radar maps shown in Fig. 8a-c. The results demonstrated that with prolonged storage, the sourness of blueberries decreased significantly, while bitterness and astringency increased markedly. This indicated that organic acids and other nutrients in blueberries were consumed by respiration or altered by microbial metabolism during storage, resulting in fruit decay and an obvious decline in taste profile quality. After 15 days of storage, blueberries preserved in the PANAM@ZIF-8@CV/PVA group showed the smallest fluctuation in sourness intensity. This confirmed that the long-term sustained release of CV reduced the respiration rate, effectively retarded the consumption of organic acids, and maintained their content, which is consistent with the above results for TA content. In addition to effectively retaining sourness, blueberries stored under this condition exhibited significantly lower bitterness, astringent aftertaste, and bitter aftertaste, together with increased umami intensity. These results further verified that the long-term sustained release of CV effectively inhibited the growth of spoilage microorganisms and reduced the formation of off-flavors (Sun et al., 2021; Zhang, Pan, & Tu, 2022). The relatively high astringency in the PANAM@ZIF-8@CV/PVA group can be explained by the excellent preservation of endogenous polyphenols (e.g., anthocyanins) in blueberries. Meanwhile, as a monoterpenoid phenol, CV itself also contributed a slight astringent sensation upon long-term sustained release (Medina-Jaramillo et al., 2020).

**Fig. 8:**
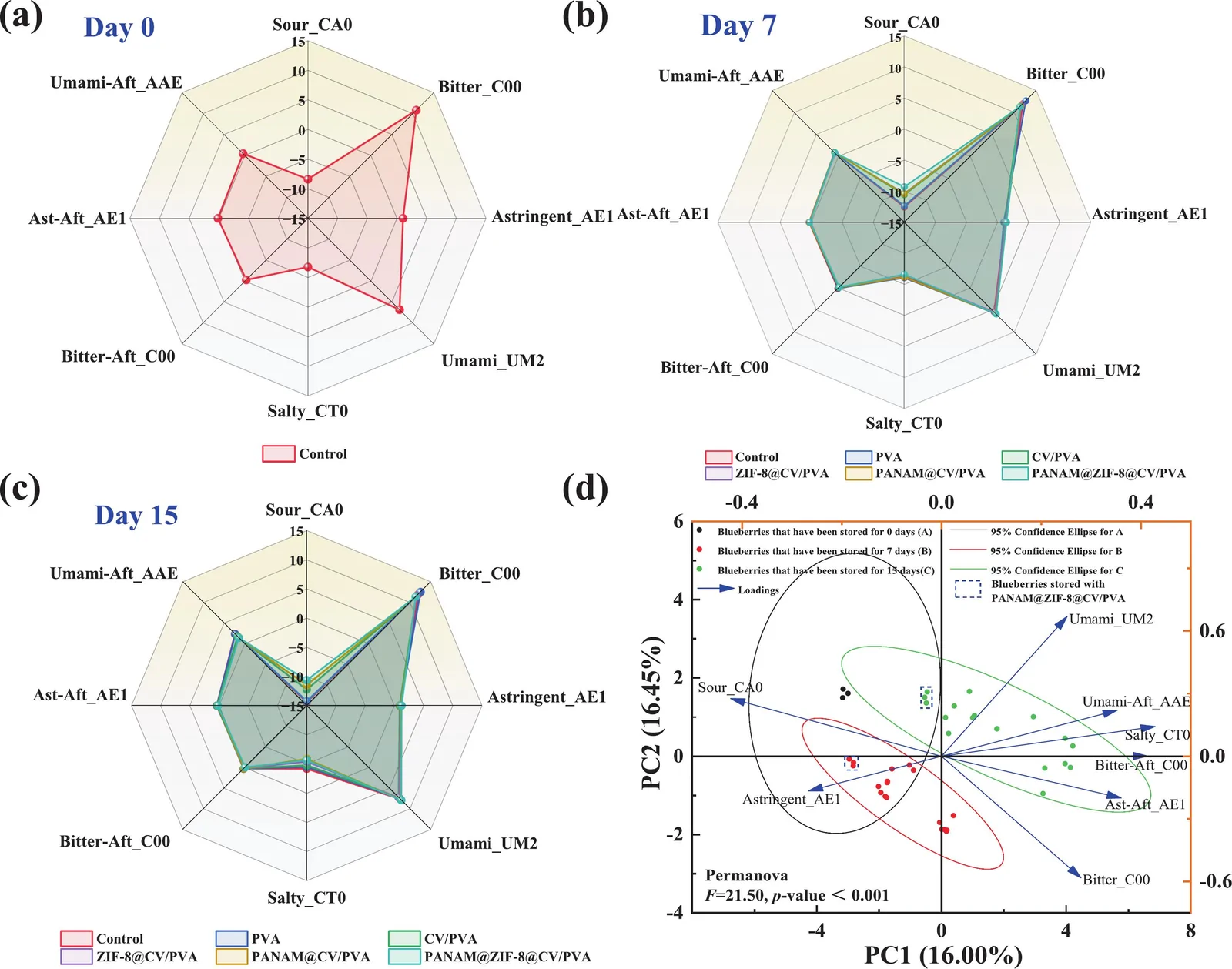
Radar charts from electronic tongue analysis of blueberries treated with different composite pads at storage Day 0 (a), Day 7 (b) and Day 15 (c); (d) PCA biplot of sensory profiles of blueberries treated with different preservation pads after 0, 7 and 15 days of storage. Triplicate samples were included for each treatment, and 95% confidence ellipses were plotted for each storage group.

As shown in Fig. 8d, the first two principal components of the PCA biplot explained 77.45% of the total variance in sensory attributes, with PC1 and PC2 accounting for 61.00% and 16.45%, respectively. Samples from different storage periods were clearly separated along the PC1 axis: fresh blueberries (Day 0) clustered on the negative side of PC1, while samples stored for 7 and 15 days gradually shifted toward the positive PC1 direction, indicating that storage duration was the dominant factor driving the changes in sensory profiles. Within the same storage period, the control group exhibited the largest shift along PC1 from Day 7 to Day 15, indicating that blueberries without packaging protection underwent the most drastic changes in taste profile over storage time (Fig. S4) (Huang et al., 2026). However, compared with other pad treatments, blueberries treated with PANAM@ZIF-8@CV/PVA (indicated by blue dashed boxes in Fig. 8d; full dataset provided in Fig. S4) were positioned closer to the fresh group along both PC1 and PC2 axes after 7 and 15 days of storage, indicating that this treatment effectively mitigated sensory deterioration and better preserved the original taste profile of fresh blueberries. PERMANOVA results (*F* = 21.50, *p* < 0.001) further verified that the overall sensory profiles among different groups were extremely significantly different. This effect could be attributed to the efficient combination of PANAM and ZIF-8, which prolonged the long-term sustained release of CV. The multiple synergistic effects inhibited taste deterioration caused by microbial metabolism. These results are consistent with the above findings, demonstrating the promising application potential of PANAM@ZIF-8@CV/PVA in blueberry preservation.

The composite pads achieve the long-term sustained release of CV via a three-level hierarchical encapsulation structure: PVA matrix – PAN porous aerogel microspheres – ZIF-8 framework.

Inner layer: The porous metal-organic framework of ZIF-8 anchors CV molecules through hydrogen bonding and Zn^2+^ coordination, avoiding burst release at the molecular level.
Middle layer: Porous PANAM not only establishes hydrogen bonding interactions with CV and offers anchoring sites for ZIF-8 and CV, but also extends the diffusion pathway, thereby slowing down its release.
Outer layer: The crosslinked PVA pad serves as a dense macroscopic barrier, which modulates the penetration of water molecules and controls the release rate of active ingredients.

At 25 °C and 60% relative humidity, CV was released steadily and continuously within 15 days, maintaining an effective concentration for antibacterial and antioxidant activity throughout storage. Through the four synergistic functions of antibacterial activity, antioxidant activity, moisture retention, and postharvest respiration inhibition, the composite pads effectively suppressed blueberry decay, browning, water loss, and senescence, thereby achieving excellent preservation at room temperature. The detailed mechanism is illustrated in Fig. 9.

**Fig. 9:**
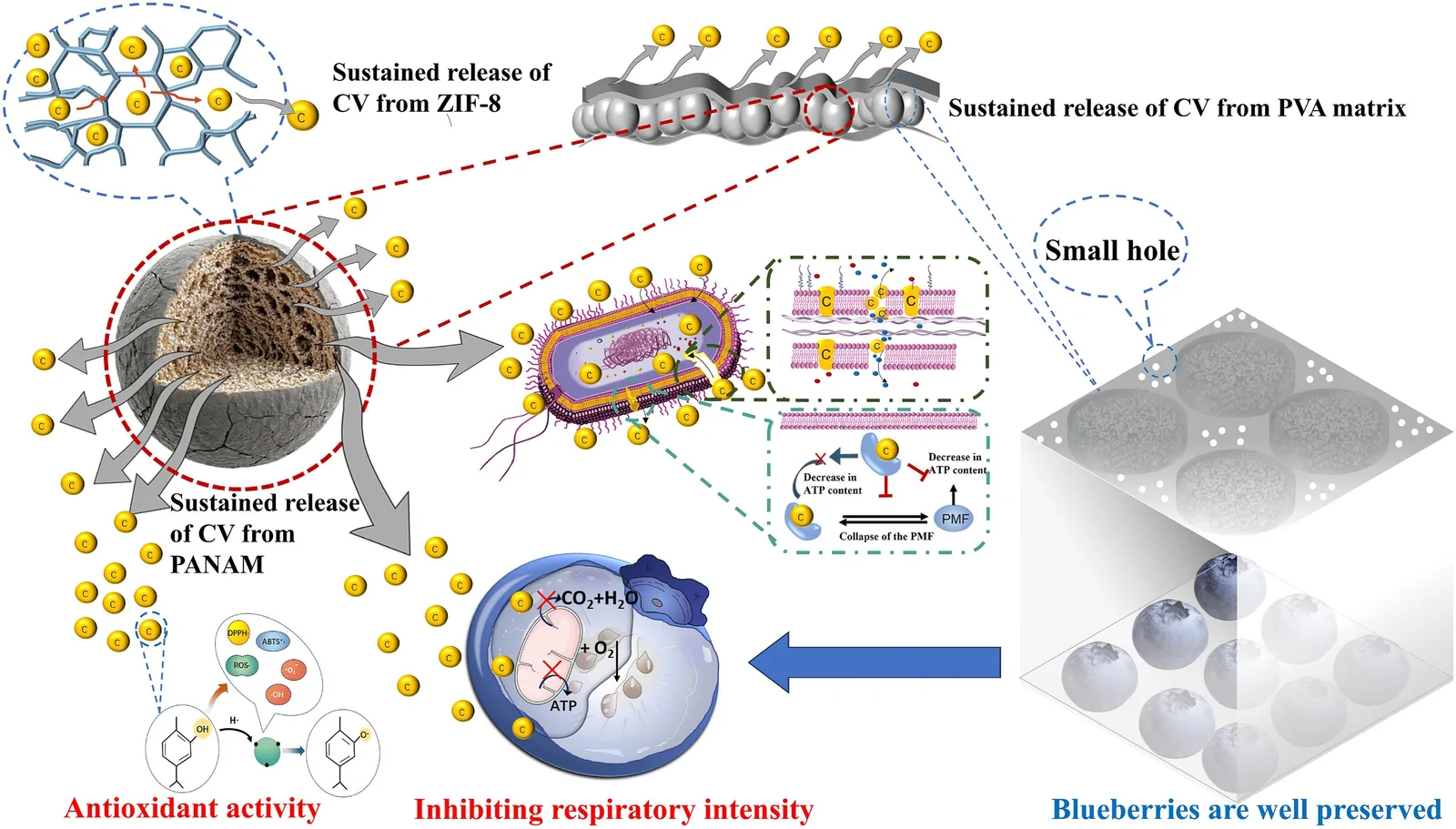
Mechanism of carvacrol sustained release and its preservation mechanism for blueberries.

## 4 Conclusion

The PANAM@ZIF-8@CV hybrid filler was successfully prepared and then blended with sodium alginate and calcium chloride crosslinked PVA to fabricate the PANAM@ZIF-8@CV/PVA composite pad. The excellent sustained release performance of CV arises from the triple synergistic effect of the composite components. Therefore, the incorporation of PANAM@ZIF-8@CV effectively reduced the UV–visible light and water vapor barrier properties of the matrix, and the resulting composite exhibited potent antioxidant activity (the maximum DPPH and ABTS radical scavenging rates were 18.06% and 78.85%, respectively) and long-lasting antibacterial activity. After 30 days of storage, the inhibition zone diameters of this composite pad against *E. coli* and *S. aureus* were 10.71 mm and 10.14 mm, respectively.

Notably, in the 15-day blueberry preservation experiment, the PANAM@ZIF-8@CV/PVA group effectively reduced the respiration intensity of blueberries (47.20 mg CO_2_·kg^−1^·h^−1^ after 15 days, versus 81.32 mg CO_2_·kg^−1^·h^−1^ for the blank group), decreased fruit weight loss (31.20% after 15 days, versus 35.7% for the blank group), and well maintained firmness (21.64 N after 15 days, versus 16.33 N for the blank group) as well as nutritional components such as VC, compared with other PVA composite pad groups. After 15 days of storage, blueberries in this group still maintained an intact appearance, and their taste profiles measured by the electronic tongue were closer to those of fresh samples on Day 0. These results demonstrate that the composite pad provides a better long-term preservation effect on blueberries as well as reveals the application potential of PANAM@ZIF-8@CV/PVA composite pads in long-acting fruit packaging.

## CRediT authorship contribution statement

**Rui Liu:** Writing – original draft, Validation, Methodology, Investigation, Formal analysis. **Yu Cheng:** Writing – original draft, Visualization, Formal analysis. **Yuanwei Peng:** Validation, Investigation, Formal analysis. **Xuelin Fan:** Funding acquisition. **Songzhi Xie:** Methodology, Formal analysis. **Baohua Liu:** Software. **Rui Qi:** Data curation. **Li Yang:** Writing – review & editing, Supervision, Methodology. **Chun Zhang:** Methodology, Investigation. **Hesheng Xia:** Methodology, Formal analysis. **Jian Wang:** Writing – review & editing, Resources, Investigation, Funding acquisition, Data curation, Conceptualization.

## Declaration of competing interest

The authors declare that they have no known competing financial interests or personal relationships that could have appeared to influence the work reported in this paper.

## Data Availability

Data will be made available on request.
