# Supplementary material for: Multifunctional PVA composite pads enabled by carvacrol-loaded ZIF-8@PAN microspheres: sustained carvacrol release and long-term blueberry preservation
Source: Food Chem X. 2026 Sep 18;39:104460. doi: 10.1016/j.fochx.2026.104460 (PMC13628657; doi:10.1016/j.fochx.2026.104460)
Supplement: Supplementary file 1 [file mmc1.docx]

**Multifunctional PVA composite pads enabled by carvacrol-loaded ZIF-8@PAN microspheres: Sustained carvacrol release and long-term blueberry preservation**

Rui Liu ^a^, Yu Cheng ^b^, Yuanwei Peng ^a^, Xuelin Fan ^c^, Songzhi Xie ^a^, Baohua Liu ^a^, Rui Qi ^a^, Li Yang ^c,*^, Chun Zhang ^c^, Hesheng Xia ^b^, Jian Wang ^a,*^

*^a^ College of Food and Biological Engineering, Chengdu University, Chengdu 610106, China*

*^b^ State Key Laboratory of Polymer Materials Engineering, Polymer Research Institute, Sichuan University, Chengdu 610065, China*

*^c^ Institute for Advanced Study, Chengdu University, Chengdu 610106, China*

*Corresponding author.

E-mail address: yanglipoly@cdu.edu.cn (L.Yang); wangjian@cdu.edu.cn (J.Wang);

**1. Characterization**

*1.1.* *Inductively coupled plasma-mass spectrometry（ICP-MS）*

Zinc content in PANAM@ZIF-8@CV, ZIF-8@CV and ZIF-8 was measured using an inductively coupled plasma-mass spectrometer (720es, Agilent, USA). *W_Zn_* stands for the mass fraction of zinc, and the corresponding value can be figured out via the Equation (S1) and Equation (S2):

$C_{Zn}(mg/kg)=\frac{C_{0}(mg/L)\times f\times V_{0}(mL)\times10^{-3}}{m(g)\times10^{-3}}$ (S1)

$W_{Zn}(\%)=\frac{C_{Zn}(mg/kg)}{10^{6}}\times100\%$ (S2)

Where, *V_o_* is a constant volume, *C_o_* is the Element concentration of Zn and *f* is the dilution ratio.

*1.2 Carvacrol (CV) loading content and CV encapsulation efficiency of different fillers*

Different composite fillers were dispersed in 10 mL of methanol, and the mixture was treated with ultrasonication for 40 min followed by 2 min of ultrasonic cell grinding with circulating water cooling. The filtered solution was centrifuged at 5366 × g and 4 °C for 5 min, and the supernatant was then transferred into a centrifuge tube for subsequent use. After blank correction, the absorbance of the sample solution was determined at a wavelength of 275 nm. Finally, the CV loading content and encapsulation efficiency in different fillers was calculated according to the standard calibration curve of CV- methanol which can be seen in Equation S3 and Equation S4. Especially, *W_t_* represents the actual loading mass of CV (mg), *W_m_* represents the sampling mass of different composite fillers, while *W_0_* refers to the theoretical loading mass of CV (mg), All tests were repeated three times, and the average value was taken as the final result.

$Carvacrol loading content (\%)\text{=}\frac{W_{t}}{W_{m}}\times100\%$ (S3)

$Carvacrol encapsulation efficiency (\%)\text{=}\frac{W_{t}}{W_{0}}\times100\%$ (S4)

*1.3. FTIR spectrum of different fillers*

Fourier transform infrared (FTIR) spectroscopic analysis of various fillers was performed using an infrared spectrometer (Spectrum 2, PerkinElmer, USA) via the KBr pellet pressing method.

*1.4. Thickness and optical properties of different composite pads*

The thickness of the different PVA composite pads were examined by using the thickness gauge Five points of each film were randomly selected to measure thickness The optical pictures of PVA composite pads (a circle with a diameter of 2.5 cm) were also captured using a mobile phone.

*1.5. Statistical analysis*

The data were presented as the mean ± standard deviation. The statistical analysis was performed by using SPSS software (version 27.0, SPSS Inc., USA) with a one-way analysis of variance (ANOVA) approach followed by Duncan's multiple range test to determine the statistical significance at *p*＜0.05.

**2. Results and discussion**

*2.1. The* *loading and encapsulation efficiency of CV in different fillers*

The loading content and encapsulation efficiency of carvacrol in the three composite fillers (PANAM@ZIF-8@CV, PANAM@CV, and ZIF-8@CV) exhibited obvious differences, as listed in Table S1. Among all samples, ZIF-8@CV presented the highest loading rate (21.19%) and encapsulation rate (52.98%), which were significantly higher than those of PANAM@CV and PANAM@ZIF-8@CV. Specifically, the loading rate and encapsulation rate of PANAM@CV were 5.29% and 13.22%, respectively, while PANAM@ZIF-8@CV only showed a loading rate of 4.28% and an encapsulation rate of 10.71%. When the addition amount of PANAM@ZIF-8@CV was fixed at 18 wt% in the composite pads, the filler contents of other PVA composite pads were calculated to keep the total CV content consistent across all groups. The calculated filler proportions were 14.56 wt% for PANAM@CV/PVA, 3.64 wt% for ZIF-8@CV/PVA, and 0.77 wt% for CV/PVA. The detailed data are presented in Table S2.

The lower loading content and encapsulation efficiency of PANAM@ZIF-8@CV and PANAM@CV can be attributed to the microporous structure on the PANAM surface, which restricts the loading capacity of CV. Although the loading and encapsulation levels of carvacrol in the PANAM-based microsphere system are relatively low, this feature endows the system with better controllability and sustained release performance. The low loading content can avoid excessive aggregation and burst release of carvacrol in composite fillers, thus allowing active molecules to be released continuously at a stable rate. Furthermore, the low encapsulation efficiency implies that most CV molecules exist in an inner surface-adsorbed state. Difficulty in encapsulation also leads to hindered release behavior, which is conducive to the slow diffusion of CV in the food preservation environment.

**Table S1**. The loading and encapsulation efficiencies of CV in different fillers

| Sample | CV loading content (wt%) | CV encapsulation efficiency (%) |
| --- | --- | --- |
| PANAM@ZIF-8@CV | 4.28 ± 0.48 | 10.71 ± 1.19 |
| PANAM@CV | 5.29 ± 0.33 | 13.22 ± 0.83 |
| ZIF-8@CV | 21.19 ± 1.63 | 52.98 ± 4.08 |

**Table S2**. The filler content and thickness of different composite pads

| Sample | Filler content (wt%) | Thickness (mm) |
| --- | --- | --- |
| PVA | 0 | 0.73 ± 0.13^b^ |
| CV/PVA | 0.77 | 0.79 ± 0.13^b^ |
| ZIF-8@CV/PVA | 3.64 | 0.79 ± 0.06^b^ |
| PANAM@CV/PVA | 14.56 | 1.11 ± 0.24^a^ |
| PANAM@ZIF-8@CV/PVA | 18 | 1.17 ± 0.20^a^ |

Different lowercase letters in the same column indicate significant difference (*p*＜0.05).

*2.2. The mass content of Zn in ZIF-8, ZIF-8@CV and PANAM@ZIF-8@CV*

The zinc element contents of all ZIF-8-containing fillers were determined by ICP-MS, and the relevant results are presented in Table S3. The zinc content in PANAM@ZIF-8@CV was measured to be 3.52%. Combined with the zinc content of pure ZIF-8 (26.43%), the loading rate of ZIF-8 in PANAM@ZIF-8@CV was calculated to be 13.32%. Meanwhile, the zinc content of ZIF-8@CV was 21.30%, by which the proportion of ZIF-8 in ZIF-8@CV was deduced to be 80.59%. Accordingly, the carvacrol (CV) content in ZIF-8@CV was 19.41%, which was close to the value determined by UV-visible spectrophotometry in the previous test.

**Table S3**. The ICP-MS results of Zn element in ZIF-8, ZIF-8@CV and PANAM@ZIF-8@CV

| Sample | Element | *V_0_* (ml) | *C_o_* (mg/L) | *f* | *C_zn_* (mg/kg) | *W_zn_* (%) |
| --- | --- | --- | --- | --- | --- | --- |
| PANAM@ZIF-8@CV | Zn | 10 | 6.55 ± 0.89 | 10 | 35209.9 ± 4770.75 | 3.52 ± 0.48 |
| ZIF-8@CV | Zn | 10 | 2.13 ± 0.04 | 100 | 213033.33 ± 4164.53 | 21.30 ± 0.42 |
| ZIF-8 | Zn | 10 | 6.11 ± 0.14 | 100 | 264293.57 ± 5998.77 | 26.43 ± 0.60 |

*2.3. The FTIR spectrum of different fillers*

The FTIR results of different fillers were shown in Fig. S1a. For pure PANAM, the characteristic peaks were observed at 3450, 2943 and 2244 cm^− 1^ are attributed to O–H stretching vibrations (from absorbed moisture), C–H stretching, and C≡N stretching vibrations, respectively. After the incorporation of CV and ZIF-8@CV into PANAM, the broad band around 3450 cm^−1^ shifted slightly to 3446 cm^−1^ and 3444 cm^−1^. Combine the characteristic C≡N stretching vibration peak of PAN slightly shifted to 2243 cm^− 1^ and 2242 after CV incorporation, which strongly verifies the formation of intermolecular hydrogen bonds between the hydroxyl groups of CV and the nitrile groups of PANAM (Yang et al., 2020; Ye, Cheng, & Yu, 2019). For ZIF-8, the characteristic peaks observed at 3418, 2931, 1592, and 1142 cm ^−1^ are attributed to the O–H stretching (from adsorbed water), C–H stretching, C=N stretching, and C–N stretching vibrations of the imidazole ring, respectively After the incorporation of CV, the broad peak in the high-frequency region shifted to 3413 cm^−1^, which is primarily assigned to the O–H stretching vibration of CV. This shift is attributed to the hydrogen-bond interaction between the hydroxyl groups of CV and the ZIF-8 framework (Li, Shan, Yu, Li, & Peng, 2023).

**Fig. S1.** (a) The FTIR spectra of different composite fillers; (b) The TGA curves of different composite fillers.

*2.4. The thermal stability of different fillers*

The thermal stability of different fillers characterized by TGA was presented in Fig.S1b. Except for pure ZIF-8, the other fillers (PANAM, PANAM@CV, PANAM@ZIF-8@CV, ZIF-8@CV) show a slight mass loss below 70 °C. This can be ascribed to the thermal detachment of fine particles on PANAM, together with the escape of adsorbed moisture and surface‑adsorbed CV from porous PANAM and ZIF-8. An obvious mass loss of ZIF-8@CV starts at 160 °C, which is caused by the volatilization of CV loaded inside the framework. This result is consistent with the conclusions reported in previous studies (Altan, Aytac, & Uyar, 2018). Above 400 °C, ZIF-8 and ZIF-8@CV gradually decompose, owing to the cleavage of coordination bonds in the ZIF-8 framework and the subsequent collapse and thermal decomposition of the crystalline structure, leading to a continuous mass loss (Li, Wang, Chen, Sun, & Tang, 2025). At 800 °C, the final residual mass of ZIF-8 is 66.7%, whereas that of ZIF-8@CV is 47.6%. The lower residual value of ZIF-8@CV is attributed to the relatively high volatility of the loaded CV (Li et al., 2023).

For PANAM, PANAM@CV, and PANAM@ZIF-8@CV, a rapid mass loss occurs at 270–290 °C, which is attributed to the thermal cleavage of the PANAM main chains and the cyclization and dehydrogenation of cyano groups (-CN). The earlier onset of rapid mass loss in PANAM@ZIF-8@CV can be ascribed to the abundant Zn^2+^ sites, which reduce the activation energy for the cleavage of PANAM main chains and exert a catalytic effect on the thermal decomposition of PANAM (Chiang & Chin, 2022; Lee, Li, Yoo, & Kim, 2021). At 800 °C, PANAM@CV shows the lowest residual mass, owing to the complete volatilization of carvacrol (CV). The residual mass of PANAM@ZIF-8@CV (36.8%) is slightly higher than that of pure PANAM (36.5%). Although ZIF-8 and ZIF-8@CV exhibit higher final residual masses, ZIF-8 is encapsulated inside the pores of PANAM microspheres. The dominant mass loss in the system arises from the outermost PANAM component, combined with the catalytic degradation of PANAM main chains by Zn^2+^, which accounts for the above result.

**Fig. S2.** High-resolution C narrow scans of ZIF-8 (a), PANAM@CV (b) and PANAM@ZIF-8@CV (c), respectively; High-resolution N narrow scans of ZIF-8 (d), PANAM@CV (e) and PANAM@ZIF-8@CV (f), respectively

*2.5. Macro/micro surface morphology and thickness of various composite pads*

The surface SEM images of all composite pads are presented in Fig. S3 (a,a′,b,b′,c,c′,d,d′,e,e′). Pure PVA exhibited a relatively smooth surface covered with nanoparticles, which were derived from sodium alginate, calcium ions, or residual calcium chloride. The CV/PVA sample showed a similarly smooth surface, while the deposited particles displayed a slightly swollen morphology due to the adsorption of CV onto the PVA surface. The ZIF-8@CV/PVA surface presented obvious regular cubic crystal structures, confirming the successful incorporation of ZIF-8. However, ZIF-8 particles were exposed on the polymer surface and exhibited severe aggregation, which might negatively affect the sustained-release behavior of CV and the overall performance of composite pads. The surface morphology of PANAM@CV/PVA was comparable to that of PANAM@ZIF-8@CV/PVA, both showing distinct surface protrusions. This finding indicated that PANAM@CV could be well dispersed and embedded within the PVA matrix, which was beneficial to the long-term sustained release of CV. In addition, a small number of microparticles could still be observed on the surface, which were likely attributable to sodium alginate and residual calcium chloride As observed from the macroscopic images (Fig. S2f), the microspheres were uniformly embedded in the PANAM@ZIF-8@CV/PVA and PANAM@CV/PVA composites with a relatively homogeneous dispersion state. By contrast, the other three PVA composite pads (neat PVA, CV/PVA, and ZIF-8@CV/PVA) exhibited relatively smooth surfaces. Compared with the two microsphere-containing composite pads, the three groups without microspheres presented better transparency, which was consistent with the UV–Vis transmittance results described in the main text.

The thicknesses of different composite pads are summarized in Table S1. Compared with neat PVA, the thicknesses of PANAM@CV/PVA and PANAM@ZIF-8@CV/PVA increased significantly after the addition of microspheres (*p* < 0.05). This phenomenon was attributed to the fact that microspheres with a diameter of approximately 500 μm dispersed in the matrix during the preparation of composite pads. They occupied extra three-dimensional space via the physical space-occupying effect, thereby greatly increasing the overall thickness of the films. In comparison, the thicknesses of CV/PVA and ZIF-8@CV/PVA showed no significant difference from that of neat PVA (*p* > 0.05), indicating that the incorporation of nano- and molecular-scale components exerted a negligible influence on the thickness of composite pads.

**Fig. S3.** Surface FESEM images of PANAM@ZIF-8@CV/PVA (a, a′), PANAM@CV/PVA (b, b′), ZIF-8@CV/PVA (c, c′), CV/PVA (d, d′), PVA (e, e′) at different magnifications. (f) Macro surface morphology of different composite pads.

**Table S4.** Fitting results of the kinetic models of the release curves of different composite pads

| Kinetic model | Sample | k | n | R^2^ |
| --- | --- | --- | --- | --- |
| Zero-order | CV/PVA | 0.0008 | / | 0.6242 |
|  | ZIF-8@CV/PVA | 0.0009 | / | 0.8151 |
|  | PANAM@CV/PVA | 0.0013 | / | 0.7622 |
|  | PANAM@ZIF-8@CV/PVA | 0.0012 | / | 0.8443 |
| First-order | CV/PVA | 0.0027 | / | 0.6885 |
|  | ZIF-8@CV/PVA | 0.0022 | / | 0.8632 |
|  | PANAM@CV/PVA | 0.0025 | / | 0.8245 |
|  | PANAM@ZIF-8@CV/PVA | 0.0022 | / | 0.9027 |
| Higuchi | CV/PVA | 0.0158 | / | 0.7926 |
|  | ZIF-8@CV/PVA | 0.0173 | / | 0.9352 |
|  | PANAM@CV/PVA | 0.0254 | / | 0.8997 |
|  | PANAM@ZIF-8@CV/PVA | 0.0234 | / | 0.9204 |
| Ritger-peppas (Q ≤ 60%) | CV/PVA | / | / | / |
|  | ZIF-8@CV/PVA | 0.3604 | 0.1220 | 0.9701 |
|  | PANAM@CV/PVA | 0.0018 | 0.2514 | 0.9705 |
|  | PANAM@ZIF-8@CV/PVA | 0.1576 | 0.2350 | 0.9269 |

**Fig. S4.** PCA biplot of blueberry samples under different storage days and PVA pad treatments (based on group average values).

**References**

Altan, A., Aytac, Z., & Uyar, T. (2018). Carvacrol loaded electrospun fibrous films from zein and poly(lactic acid) for active food packaging. *Food Hydrocolloids, 81*, 48-59. https://doi.org/10.1016/j.foodhyd.2018.02.028.

Chiang, Y.-C., & Chin, W.-T. (2022). Preparation of Zeolitic Imidazolate Framework-8-Based Nanofiber Composites for Carbon Dioxide Adsorption. *Nanomaterials*, *12(9)*, 1492. https://doi.org/10.3390/nano12091492.

Lee, J.-H., Li, S., Yoo, J.-B., & Kim, Y.-J. (2021). Effects of Various Transition Metals on the Thermal Oxidative Stabilization of Polyacrylonitrile Nanofibers. *Journal of Inorganic and Organometallic Polymers and Materials, 31*(8), 3368-3377. https://doi.org/10.1007/s10904-021-01954-x.

Li, H., Wang, S., Chen, J., Sun, M., & Tang, H. (2025). Preparation and characterization of thymol-loaded ZIF-8/κ-carrangeenan/Zein composite film as active food packaging with enhanced functional properties for blueberry preservation. *Carbohydrate Polymers, 356*, 123406. https://doi.org/10.1016/j.carbpol.2025.123406.

Li, Y., Shan, P., Yu, F., Li, H., & Peng, L. (2023). Fabrication and characterization of waste fish scale-derived gelatin/sodium alginate/carvacrol loaded ZIF-8 nanoparticles composite films with sustained antibacterial activity for active food packaging. *International Journal of Biological Macromolecules, 230*, 123192. https://doi.org/10.1016/j.ijbiomac.2023.123192.

Yang, L., Yang, J., Qin, X., Kan, J., Zeng, F., & Zhong, J. (2020). Ternary composite films with simultaneously enhanced strength and ductility: Effects of sodium alginate-gelatin weight ratio and graphene oxide content. *International Journal of Biological Macromolecules, 156*, 494-503. https://doi.org/10.1016/j.ijbiomac.2020.04.057.

Ye, H., Cheng, J., & Yu, K. (2019). In situ reduction of silver nanoparticles by gelatin to obtain porous silver nanoparticle/chitosan composites with enhanced antimicrobial and wound-healing activity. *International Journal of Biological Macromolecules, 121*, 633-642. https://doi.org/10.1016/j.ijbiomac.2018.10.056.
